# Energy Transfer Sensitization of Luminescent Gold Nanoclusters: More than Just the Classical Förster Mechanism

**DOI:** 10.1038/srep35538

**Published:** 2016-10-24

**Authors:** Eunkeu Oh, Alan L. Huston, Andrew Shabaev, Alexander Efros, Marc Currie, Kimihiro Susumu, Konrad Bussmann, Ramasis Goswami, Fredrik K. Fatemi, Igor L. Medintz

**Affiliations:** 1 Optical Sciences Division Code 5600, U.S. Naval Research Laboratory, Washington, DC 20375, USA; 2 Sotera Defense Solutions, Inc. Columbia, MD 21046, USA; 3 Center for Computational Material Science Code 6390, U.S. Naval Research Laboratory, Washington, DC 20375, USA; 4 Materials and Sensors Branch Code 6361, U.S. Naval Research Laboratory, Washington, DC 20375, USA; 5 Multifunctional Materials Code 6351, U.S. Naval Research Laboratory, Washington, DC 20375, USA; 6 Center for Bio/Molecular Science and Engineering Code 6900, U.S. Naval Research Laboratory, Washington, DC 20375, USA

## Abstract

Luminescent gold nanocrystals (AuNCs) are a recently-developed material with potential optic, electronic and biological applications. They also demonstrate energy transfer (ET) acceptor/sensitization properties which have been ascribed to Förster resonance energy transfer (FRET) and, to a lesser extent, nanosurface energy transfer (NSET). Here, we investigate AuNC acceptor interactions with three structurally/functionally-distinct donor classes including organic dyes, metal chelates and semiconductor quantum dots (QDs). Donor quenching was observed for every donor-acceptor pair although AuNC sensitization was only observed from metal-chelates and QDs. FRET theory dramatically underestimated the observed energy transfer while NSET-based damping models provided better fits but could not reproduce the experimental data. We consider additional factors including AuNC magnetic dipoles, density of excited-states, dephasing time, and enhanced intersystem crossing that can also influence ET. Cumulatively, data suggests that AuNC sensitization is not by classical FRET or NSET and we provide a simplified distance-independent ET model to fit such experimental data.

Förster resonance energy transfer (FRET) is a powerful technique for probing small changes in separation distance between donor and acceptor fluorophores^1^,^2^,^3^,^4^,^5^. Although FRET has typically relied on dyes and/or fluorescent proteins as both donors and acceptors, the development of fluorescent nanoparticles has greatly expanded the potential utility^6^,^7^,^8^,^9^,^10^. FRET imaging and biosensing applications have directly benefited from a growing library of donor/acceptor materials including dye-doped nanoparticles, upconversion materials, inorganic chelates and especially luminescent semiconductor quantum dots (QDs). Critically, an understanding of the underlying photophysical processes involved provides a predictive capability for rationally designing new FRET configurations^7^,^11^,^12^,^13^,^14^. Within the broad FRET application space, however, the lack of small-robust and biocompatible donor/acceptor materials that emit in the near-IR (*ca*. 700–1000 nm) remains a significant roadblock towards fully accessing this biologically important portion of the spectrum^7^.

The recent development of luminescent near-IR emitting gold nanoclusters (AuNCs) has generated strong interest and may represent a potential solution to this issue^15^,^16^,^17^,^18^,^19^. This interest is driven by their unique characteristics including facile chemical synthesis, excellent biocompatibility, effective chemistries for bioconjugation (*i.e*., Au-S interactions), small size (<3 nm diameter) and, most importantly, strong emission in the near-IR tissue transparency window (700–900 nm)^16^,^17^,^18^,^20^,^21^. The mechanism(s) by which AuNCs luminesce are, however, complex and still not fully understood. Zheng *et al*. describes two classes of luminescent AuNCs that are differentiated as “few atom” NCs, consisting of clusters with diameters <1.2 nm, and “few nm” NCs, characterized by diameters between 1.5 nm and 3 nm^18^. Luminescence of the smaller NCs is believed to arise from quantum-confinement effects within the core, while emission for the “few nm” materials is believed to be involved with ligand-metal charge transfer (LMCT) processes^16^,^18^,^22^. The AuNCs described here appear to have properties of both classes given their physical size, near-IR emission and luminescence lifetimes that approach 1 μs.

A rapidly growing number of energy transfer (ET) configurations exploring AuNCs have also begun to appear. Cheng *et al*. were the first to report a 40% sensitization of near-IR emitting 1.8 nm diameter tiopronin-protected AuNCs by (Ru(bpy)_3_)^2+^ donors *via* dynamic quenching in an aqueous solution, although no underlying ET process was defined^23^. Montali described enhanced near-IR emission from 1.8 nm AuNCs following sensitization by modified pyrene donor chromophores that were introduced during synthesis as a ligand replacement^24^. Both FRET (1/*R*^6^ distance dependence where *R* is the donor-to-acceptor separation distance)^4^ and nanosurface energy transfer (NSET, 1/*R*^4^ dependence) models^25^ were reported to be compatible with quenching observed in these assemblies. Aldeek coupled AuNCs to a series of QDs by exploiting surface ligand chemistry and observed substantial QD quenching, although no concomitant AuNC sensitization was observed^26^. Energy transfer from the QD in this configuration was partially ascribed to FRET dipole-dipole coupling along with other putative non-Förster processes. Pradeep’s group synthesized glutathione-stabilized Au_25_ clusters which were further functionalized by attaching dansyl chromophores to the cluster core^27^. The experimental data was evaluated using both FRET and NSET formalisms with the observed dynamics primarily attributed to FRET. A subsequent report from the same group utilized the protein lactoferrin as a stabilizer to synthesize AuNCs and characterized the ET from the proteins fluorescent residues to the clusters using FRET^28^. Raut and colleagues reported use of bovine serum albumin (BSA)-protected AuNCs as both resonance energy transfer (RET) acceptors and donors with the organic dyes Pacific Blue and Dylight 750, respectively^29^. Pu *et al*. reported on intracellular detection of mercury ions in a configuration using blue-emitting silsesquioxane polyhedral oligomeric donors to sensitize BSA-encapsulated 700 nm emitting AuNC acceptors (0.8 nm diameter, 25 atoms); sensitization was specifically ascribed to a FRET process in this case^30^. Similarly, Qin reported quenching interactions between glutathione-capped 2.4 nm AuNCs and gold nanorods as FRET as well^31^.

Beyond just characterizing the ET interactions and observing sensitization in the experimental donor-AuNC systems, publications are also starting to accumulate where FRET theory is directly applied to the data to extract further physical information about a given configuration. For example, Russell *et al*. estimated a tryptophan-AuNC donor-acceptor separation distance using FRET formalism for human serum albumin- (HSA) stabilized AuNCs^32^. This information was then used to correlate where the clusters were nucleating on the protein structure. Raut *et al*. extracted distances from tryptophan to the AuNC emission center based on FRET for both BSA and HSA-stabilized AuNCs^33^. Similarly, Bain studied the role of different AuNC acceptor surface capping ligands as they interacted with CdTe QD donors and, although no AuNC sensitization was overtly seen, they utilized FRET formalism to extract putative QD donor-AuNC acceptor distances^34^. Other examples assigning FRET^35^ or some other undefined ET processes^36^ for tryptophan sensitization of AuNCs can also be found. In contrast to the above examples, Liu reported quenching of dye molecules in the presence of glutathione-stabilized Au_25_ clusters which could be recovered after dissociating the clusters^37^. DNA-templated AuNCs have also been assembled with a spectrally favorable SYBR Green I donor dye present in the DNA, however, no ET was observed from the intercalating dye to the clusters^38^. AuNCs have also been reported to function as FRET donors for dyes^39^ and NSET donors to non-emissive gold nanoparticles (AuNPs)^40^. Other reports describe electron transfer as the underlying transfer mechanism with examples including quenching of BSA-stabilized Au_25_ clusters by graphene oxide and use of Au_10,15,18,25_ glutathione-stabilized clusters as light harvesting antennae for subsequent interactions with the electroactive dye methyl viologen^41^,^42^. Interestingly, QD interactions with graphene have been previously ascribed to a FRET-like process but with a 1/*R*^4^ donor-acceptor distance dependence due to the acceptor’s 2-dimensional nature^43^,^44^,^45^.

Clearly, AuNC sensitization by ET is a repeatable phenomenon, however, the difference in suggested governing ET mechanisms likely does not arise from a wide diversity of underlying ET process(es), but rather reflects the general lack of understanding that currently exists regarding ET to- and especially sensitization of AuNCs. Here, we investigate the ET characteristics of luminescent AuNCs as acceptors with a series of structurally and functionally distinct luminescent donor systems, including Cy3 and Rhodamine-Red (R-Red) molecular dyes, Ru- and Tb-metal ion complexes and two different QDs using clearly defined-stoichiometric conjugates, see Fig. 1 and Supplementary Information (SI) Figs S1 and 2. This represents the largest systematic study of AuNC sensitization with different classes of donor. Moreover, AuNC sensitization by the QDs and Tb(chelate) are the first descriptions of this type of phenomena for those particular materials in this ET configuration. Experimental data is then used to determine the efficiencies of the intermolecular ET processes for initial comparison to predictions made using FRET and dye-metal damping theories. In direct contrast to many of the previous reports, we find that none of the models fit the data completely, strongly suggesting that AuNC sensitization does not occur *via* traditional FRET or NSET as we currently understand it. We consider the role of additional factors that take into account the unique electronic properties of ultra-small luminescent AuNCs including their exceptionally high density of excited states, rapid dephasing time and strong electron confinement as well as the role of the cluster’s paramagnetic properties. Lastly, we provide a simplified distance independent ET model to fit such experimental data.

## Results

### Synthesis and Characterization of Luminescent AuNCs

**Figure 1:**
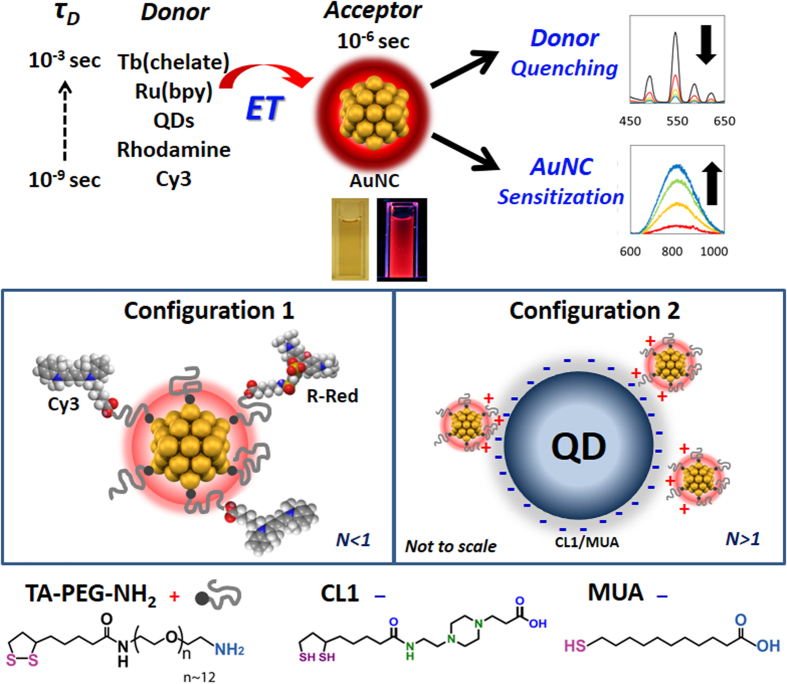
Experimental configuration and materials. **(Top)** Schematic of the ET experimental formats combining various donors with luminescent AuNCs and images of AuNC aqueous solutions under ambient light (left) and 365 nm UV lamp (right). **(Middle)** Schematic diagram of the two different donor-acceptor configurations utilized. Configuration 1: (AuNC)_N_/Dyes (*N* = 0.08~0.6) and Configuration 2: (AuNC)_N_/QDs (*N* = 2~15 for QD545 and *N* = 5~40 for QD625). **(Bottom)** Chemical structures of TA-PEG-NH_2_ used to cap the AuNCs and CL1/MUA used to cap the QDs.

**Figure 2:**
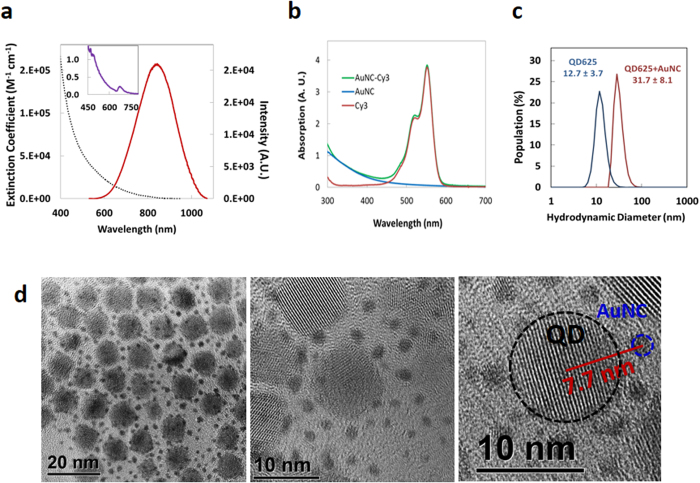
Characterization of selected AuNC materials. **(a)** Absorption and PL spectra of AuNCs. Inset presents excitation spectrum highlighting the structural features. **(b)** Representative absorption spectra showing the deconvolved AuNC and Cy3 components for AuNC/Cy3 *N* = 0.08. **(c)** DLS measurements of QD625 (blue) and (AuNC)_N_/QD625 (N = 40) (red) in water by population profile. **(d)** TEM micrographs of (AuNC)_N_/QD625 (*N* = 40). The core sizes of QD545 and QD625 were determined to be 4.0 ± 0.29 nm and 9.2 ± 0.81 nm, respectively. Note, not all the AuNC’s surrounding each QD are visualized in a given TEM as many are obscured underneath the QDs or not in the correct plain of focus.

**Figure 3:**
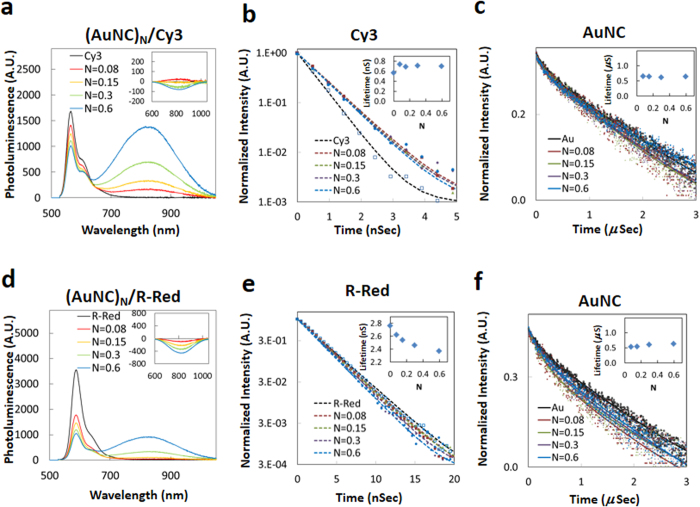
Steady state and time-resolved data from conjugating Cy3 and R-Red donor to AuNC acceptors. PL changes from donors titrated with a mixture of “*N*” equivalents of AuNCs. **(a,d)** PL changes of (AuNC)_N_/Cy3 system and (AuNC)_N_/R-Red, respectively, excited at 470 nm. Inset shows net PL spectral changes from AuNCs representing the sensitized acceptor intensity component. **(b,e)** Excited state lifetime measurements of Cy3 and R-Red in (AuNC)_N/_/dye systems, respectively. Inset plots the conjugation ratio (*N*) dependence of the donor’s averaged lifetimes. **(c,f)** Excited state lifetime measurements of AuNC in (AuNC)_N_/dye systems. Inset shows the conjugation ratio (*N*) dependence of the AuNC averaged lifetimes.

**Figure 4:**
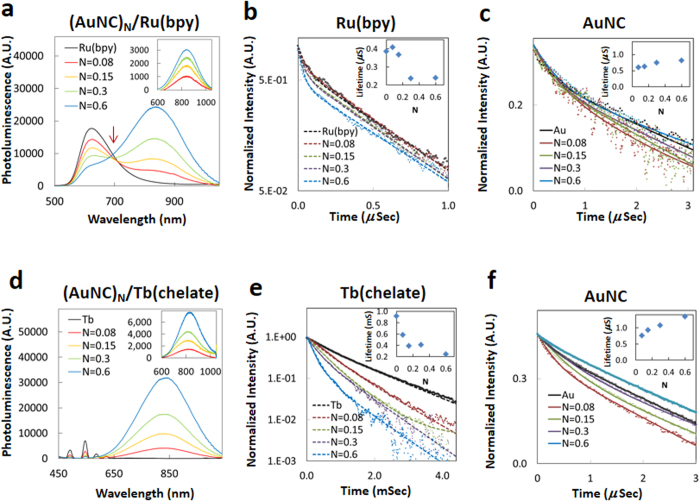
Steady state and time-resolved data from conjugating Ru(bpy) and Tb(chelate) donor to AuNC acceptors. PL changes from donors titrated with a mixture of “*N*” equivalents of AuNCs. **(a,d)** PL changes of (AuNC)_N_/Ru(bpy) system and (AuNC)_N_/Tb(chelate), respectively, excited at 470 nm. Inset is the net PL spectral changes from AuNCs representing the sensitized acceptor intensity component. **(b,e)** Excited state lifetime measurements of Ru(bpy) and Tb(chelate) in (AuNC)_N_/donor systems, respectively. Inset plots the conjugation ratio (*N*) dependence of the donor’s averaged lifetimes. In e, the apparent rise-time differences are attributed to the slow CS-124 to Tb to AuNC transfer processes and are not considered for excited state lifetime calculation. **(c,f)** Excited state lifetime measurements of AuNC in (AuNC)_N_/donor systems. Inset shows the conjugation ratio (*N*) dependence of the AuNC averaged lifetimes.

**Figure 5:**
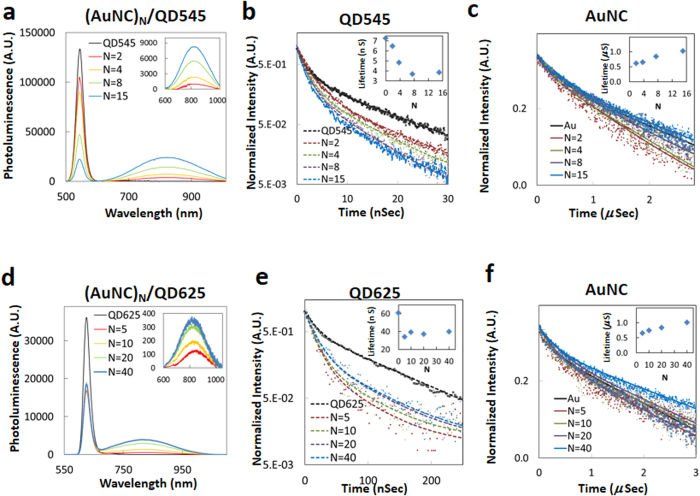
Steady state and time-resolved data collected from the assemblies of QD545 and QD625 donors with AuNCs. **(a,d)** PL changes from (AuNC)_N_/QD545 and (AuNC)_N_/QD625 with the indicated “*N*” equivalents of AuNCs. Inset is the net PL spectral changes from the AuNCs representing the sensitized acceptor intensity. **(b,e)** Excited state lifetime measurements of QD545 and QD625 in the (AuNC)_N_/QD systems, respectively. Inset shows the conjugation ratio (*N*) dependence of the donor’s averaged lifetimes. **(c,f)** Lifetime monitoring of AuNC in these systems. Inset plots the conjugation ratio (*N*) dependence of the AuNC averaged lifetimes.

We previously reported the synthesis of biocompatible, near-IR luminescent AuNCs in water using poly(ethylene glycol) (PEG)-dithiolane ligands terminated with either a carboxyl, amine, azide, or methoxy group^46^. In-depth characterization showed the AuNCs were easily delivered to and well tolerated by cells while providing robust luminescence in the near-IR tissue transparency window^46^. General synthetic details are described in the Supplementary Methods. In order to suppress the growth of the AuNC core within the cluster regime, excess ligands were added during synthesis to obtain 1.5 nm AuNCs. TEM measurements confirmed that the AuNC size was 1.5 ± 0.30 nm without any size purification, and STEM confirmed the NCs displayed heavy atoms (Au) which appear as bright spots in the STEM images (TEM in Fig. 2d, STEM in Supplementary Fig. S3). We note that the clusters’ optical and physical properties such as quantum yields (*Φ*) did not change over a period of ~2 years and were not affected by the presence of different buffers (SI Fig. S4) similar to the results presented previously^46^. The maximum number of bidentate thioctic acid (TA)-PEG-NH_2_ ligands on the AuNC surface is estimated at ~22 allowing us to potentially attach a similar number of dyes around the nanocluster periphery, see Fig. 1 and Supplementary Methods for the calculation^46^,^47^. The relevant AuNC photophysical properties including absorption maxima, emission peaks, excited state lifetimes, radiative and non-radiative decay rate (*k*_*r0*_ and *k*_*nr0*_, respectively) are presented in Table 1. *Φ* values were calculated by comparing the photoluminescence (PL) intensities and absorbance to those of Rhodamine-6G and Indocyanine Green dye standards. The absorption and PL spectra of AuNCs and the donor sample set utilized are shown in Fig. S2 with the latter shown superimposed over the AuNC acceptor absorption. The AuNCs are characterized by a very broad, continuous, monotonically-decreasing absorption spectrum extending from the UV through the near-IR (Fig. 2a). Due to their small size, they do not exhibit the distinctive surface plasmon resonance band that is characteristic of AuNPs over 3 nm in diameter. Rather, the PL excitation spectrum does show a distinctive feature around 673 nm (Inset, Fig. 2a). Specific absorption/excitation features have been reported for small AuNCs, of which the origins are not well understood. Suggested sources of these features include: (1) the energy band gap between the highest occupied molecular orbital (HOMO) and the lowest unoccupied molecular orbital (LUMO) originating from a quantum confinement effect, and (2) LMCT^18^,^22^,^48^,^49^. AuNC emission spectra show a PL maximum around 820 nm with a relatively broad profile (full width at half maximum ~220 nm). *Φ* was relatively high for water-soluble AuNCs at ~6% with an average PL lifetime of ~0.75 μs when excited at 355 nm and monitored at 800 nm. Lifetime data were fit to bi-exponential decay functions (Figs 3, 4 and 5) and the measured lifetimes differed slightly depending upon the excitation/emission wavelengths used, similar to previous reports^46^,^50^. Actual PL decay dynamics are more complex than suggested by a simple bi-exponential model and could indicate a diversity of AuNCs with different photophysical characteristics due to slightly different shapes, sizes and ligand coordination geometries.

### Assembly and Characterization of AuNC-Donor Conjugates

Photophysical characteristics of the molecular dyes, metal chelates and the QD donors are summarized in Table 1. The significant physical size differences amongst the donors required the use of two different geometric assemblies: Configuration 1-a single PEGylated AuNC acceptor surrounded by varying numbers of dyes or metal chelate donors and Configuration 2-compact ligand CL1/mercaptoundecanoic acid (CL1/MUA)-capped QD donors surrounded by varying numbers of PEGylated AuNC acceptors (see Fig. 1). The acceptor-donor hybrids with different AuNC-to-donor-ratios, *N*, were achieved by controlling the reaction stoichiometry and conjugation was verified in representative samples by UV-vis absorption following purification of the conjugate with size exclusion chromatography (Fig. 2b): a full description is provided in the SI. We also confirmed formation of the (AuNC)_N_/QD625 configuration and estimated the distance between the different NPs using TEM and dynamic light scattering (DLS) measurements; these were then compared to the theoretical predictions of distance (details in Supplementary Methods and Table S1). Figure 2c shows the hydrodynamic size changes of QD625 with and without AuNC conjugation based on DLS number profile (population *vs.* size). Figure 2d shows representative TEMs of (AuNC)_N_/QD625 (*N* = 40) conjugates assembled by electrostatic attraction between the negatively charged CL1/MUA-capped QD625 and positively charged AuNC-TA-PEG-NH_2_. TEM images confirmed that more than 30 AuNCs surrounded each QD625 when assembled with a reaction stoichiometry of *N* ~ 40. Importantly, the TEM analysis did not show any QDs stacking on top of the other QDs which would arise from 3-D aggregation or crosslinking. Rather, they reflect well-separated conjugates in a 2-D packing arrangement which would arise from the dehydration of homogeneous samples as expected. The separation distance (*R*) between AuNC and QD625 was measured to be ~7.7 nm in the dehydrated TEM sample; note this is *not* the value utilized for extrapolating the experimental QD-AuNC separation. The averaged hydrodynamic distance between the AuNC and QD625 in water was determined to be ~11.7 nm by considering the hydrodynamic size of the QD (see Supplementary Table S1). The PEG 600 portion of the AUNC ligand separating them should theoretically increase the hydrodynamic radius by ~1.4 nm based on a Worm-Like-Chain (WLC) model and the remainder of the TA-PEG-NH_2_ should increase the hydrodynamic radius by another ~2.7 nm, see SI. The QD’s CL1 ligand should also increase the hydrodynamic radius by ~2 nm. Considering these values in conjunction with the core sizes of AuNC and QD625 from TEM (~1.5 nm and ~9.2 nm respectively), the theoretical separation distance between the AuNC and QD625 was extrapolated to ~10.1 nm which is close to the measured hydrodynamic size of ~11.7 nm (~16% difference). Table 2 summarizes estimated donor-acceptor separation distances for all assemblies and includes minimum/maximum values to establish upper and lower limits (*i.e*. error) for the predicted ET efficiencies obtained from calculations.

### Photophysical Characterization of AuNC/Donor Assemblies

We selected three classes of donors commonly used for ET studies with significantly different physicochemical properties, electronic structures and lifetimes. In Configuration 1, the AuNC acceptors are surrounded by varying numbers of dyes or metal chelate donors (AuNC_N_/donor *N* < 1) and in Configuration 2, the QD donor is surrounded by varying numbers of AuNC acceptors (AuNC_N_/donor *N* > 1). In comparison to the use of a single donor-to-acceptor ratio, the use of different ratios improves the accuracy of the data as it surveys a wide cross-section of interactions and thus provides more insight into the underlying trends. The following provides a brief overview of results for each configuration. Where applicable, values for both donor steady state and lifetime quenching along with any apparent acceptor PL sensitization within each configuration/ratio are presented in Supplementary Table S4. Assembly concentrations and applicable ratios are also described in Conjugation of Donors to AuNC Acceptors section within the SI.

#### Cy3 and Rhodamine-Red Dye Donors

These dyes are characterized by relatively short luminescence lifetimes (<3 ns) with emission originating from their lowest excited singlet states and with lower energy, non-emissive triplet states. Figure 3a–c shows representative spectra collected from AuNCs labeled with varying Cy3 ratios along with the Cy3 and AuNC decay profiles. Steady state luminescence measurements using native AuNCs were performed under the same conditions for use as a negative control to account for the direct AuNC excitation contributions. The raw spectra show that as the ratio of AuNC/Cy3 increases, the PL intensity of the Cy3 component within the conjugates decreases while the AuNC PL at 820 nm appears to increase. In comparison to donor-only control samples, we measured a Cy3 quenching efficiency of up to 41% for *N* = 0.6 (Table S4). To extract the net AuNC sensitization signal within the conjugates, we subtracted the direct excitation contribution from the total AuNC emission in the absence of donor and present the net PL in Fig. 3a inset. The same methodology was repeated for all other relevant data shown below (Figs 4 and 5). Following this processing, no enhancement of the AuNC emission was observed and, in fact, the AuNC PL decreased slightly (~6% for *N* = 0.6, Fig. 3a-inset) which is distinctive compared to the other donor-AuNC configurations described below. Time-resolved PL profiles for Cy3 (580 nm) and the AuNCs (850 nm) are shown in Fig. 3b,c, respectively. Insets in these figures represent the amplitude-weighted average lifetimes as a function of *N*. Detailed results of the lifetime analysis for each conjugation ratio are presented in Table S2. Despite the quenching observed in the steady-state fluorescence spectra, the Cy3 lifetimes measured within the different conjugation ratios showed almost no changes except a small increase (from 0.57 ns to 0.71 ± 0.22 ns, ~25%) compared to the unconjugated Cy3. This observation is qualified by the fact that the increase is within the measurement error of our system and did not display any systematic pattern.

Similar phenomena were observed for the R-Red/AuNC conjugates (Fig. 3d–f). Steady state R-Red PL decreased by ~74% compared to the unconjugated dye for *N* = 0.6 (Table S4). No corresponding sensitized emission signal from the AuNC acceptors was observed following deconvolution and again, a similar overall slight decrease in acceptor PL was observed (Fig. 3d-inset). R-Red PL decay time decreased within the conjugate series by ~14% from 2.8 ns for the isolated dye to 2.5 ± 0.1 ns. AuNC conjugate average lifetimes were approximately 30% shorter than the unconjugated AuNCs (0.51 *vs*. 0.73 μs).

#### Ru(bpy) and Tb(chelate) Metal Donors

We next examined ET dynamics for metal ion complexes conjugated to AuNCs.The Ru(bpy) and Tb(chelate) complexes are characterized by emission from symmetry forbidden, lowest energy excited states with relatively long luminescence lifetimes of 0.39 μs and 910 μs, respectively^51^. Figure 4a–c shows representative steady state PL spectra of the Ru(bpy)-AuNC conjugates excited at 470 nm along with the deconvoluted AuNC sensitization data and the PL lifetime decay profiles for the Ru(bpy) and AuNC components, respectively. A systematic quenching of the steady state Ru(bpy) luminescence around 615 nm was observed with increasing AuNC/chelate ratio, exhibiting a maximum quenching efficiency (*E*_*Q*_, Eq. S2) of 74% for *N* = 0.6 (Table S4). The inset of Fig. 4a shows the increasingly positive deconvolved sensitization signal from the isolated AuNC component of the conjugates, with a maximum (*N* = 0.6) 60% sensitization efficiency, (*E*_*Sen*_, Eq. S4). Indeed, quenching of the Ru(bpy) along with concomitant sensitization of the AuNC PL are so pronounced in the raw data as *N* changes that a clear isosbestic point is noted at ~690 nm (indicated by arrow) while changes in their relative magnitudes inversely track each other. Changes in the PL lifetime of Ru(bpy), as a function of the conjugation ratio are shown in Fig. 4b. The average lifetime of the Ru(bpy) donor decreased with increasing AuNC/Ru(bpy) ratio, showing a 38% decrease for *N* = 0.6 while that of the AuNC acceptor increased ~8%.

Donor quenching and acceptor sensitization characteristics of the AuNC/Tb(chelate) system are summarized in Fig. 4d–f and Table S4. Tb(chelate) steady state PL intensity decreases systematically as *N* is varied between 0.08 and 0.6 (−56% to −94%). An unexpectedly large increase in the PL from the AuNCs was observed as the conjugation ratio was increased. In fact, the AuNC signal enhancement was much higher than that expected from the donor quenching measurements, suggesting additional processes could be occurring in these conjugates. In addition to the expected Tb PL lines in the visible, a bright blue emission was observed from the isolated Tb(chelate) (Fig. S6a), indicating a potential contribution associated with the chelate itself from this portion of the spectrum to the total sensitized PL from the AuNCs. To address this, we performed additional experiments in which we conjugated the isolated cs-124 chelate moiety to the AuNCs without the Tb present. For this configuration, the PL *Φ* of the AuNCs PL increased to 7–7.5% from an initial value of 5–6% (17–25% increase above initial native value). This suggested a direct ET or quantum efficiency augmentation from cs-124 to the AuNCs in addition to any ET from the Tb. After correcting and accounting for this, the recalculated sensitization efficiency from the AuNCs reached a maximum of 94% for *N* = 0.6, consistently tracking with the donor quenching measurements (Table S4). Quenching efficiencies for each discrete Tb(chelate) emission band in the AuNC/Tb(chelate) complexes were also measured and all bands showed a similar PL quenching trend to that observed for the total emission (Fig. S6b). The results of the donor and acceptor lifetime measurements are summarized in Table S2. Analogous to the Ru(bpy) assembly above, ET efficiencies observed from lifetime measurements were smaller than those obtained from steady state PL measurements. Lifetimes obtained for the Tb(chelate), measured at the primary 540 nm emission line, decreased from 910 to 240 μs as *N* varied from 0.08 to 0.6 (Table S4 and Fig. 4e). AuNC lifetimes within the conjugates increased significantly from 0.34 μs for *N* = 0.08 to 1.07 μs for *N* = 0.6 (Table S2 and Fig. 4f).

#### Quantum Dot Donors

QDs have been utilized as efficient ET donors for a variety of dye and other acceptors within various two- and three-dimensional nanoarchitectures^52^,^53^,^54^,^55^,^56^,^57^,^58^, along with AuNPs^59^,^60^,^61^,^62^,^63^,^64^, but they have almost exclusively not been shown to enhance the AuNC PL signal if present. To investigate their interactions with AuNCs, we utilized two different QDs: green emitting QD545 with a native averaged lifetime (*τ*_*D0*_) of 7.3 ns and *Φ* of 0.16 and red emitting QD625 with a *τ*_*D0*_ of 61 ns and *Φ* of 0.50. For these experiments, Configuration 2 was used where a single QD was surrounded by varying numbers of AuNCs (Fig. 1).

Figure 5a–c shows PL spectra of the AuNC/QD545 conjugates ranging from *N* = 0-to-15 along with the PL decay profiles for the QD545 and AuNC components. The inset in Fig. 5a shows enhanced AuNC luminescence after correction for the direct AuNC excitation. The steady state PL quenching of the QD545 donor reached ~83% for *N* = 15 and the corresponding AuNC luminescence sensitization efficiency appeared comparable reaching a significant 93% (Table S4). The average lifetime for the QD545 donor gradually decreased from 7.3 ns for *N* = 0 to 3.9 ns for *N* = 15, yielding a putative energy transfer efficiency of about 50% (Table S2). The average lifetime of the AuNC acceptor was initially 0.62 μs for *N* = 2 and then increased to 1.02 μs for *N* = 15 (Fig. 5c). The relevant spectral and temporal characteristics of the AuNC/QD625 conjugates are shown in Fig. 5d–f. Figure 5d shows spectra for the AuNC/QD625 conjugate ratios ranging from *N* = 0 to 40 with the inset again reflecting an enhancement of AuNC sensitized luminescence. The steady state PL of QD625 was quenched by 64% with just 5 conjugated AuNCs and remained near this value as the number of AuNCs continuously increased thereafter to 40. The average QD625 donor lifetime decreased almost 50% from 61 ns for *N* = 0 to 34 ns for *N* = 5 and then averaged 38.7 ± 1.5 ns for the remaining ratios while the AuNC average lifetime concomitantly increased from 0.73 to 1.01 μs across this range.

### Energy Transfer Models

To establish a plausible mechanism for the observed photophysical phenomena between the various donors and the AuNC acceptors, we consider three possible energy transfer models: (1) FRET; (2) NSET; and (3) Nanovolume Energy Transfer (NVET) which is modified from Persson’s volume damping theory; pertinent details of each mechanism are discussed below or in the SI. A generalized expression for ET efficiency, *E*_*ET*_, can be written as follows:


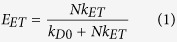


where *k*_*D0*_ is the decay rate of the donor in the absence of acceptor and is a summation of the radiative (*k*_*r0*_) and nonradiative decay (*k*_*nr0*_) rates (*k*_*D0*_* *=* k*_*r0*_* *+* k*_*nr0*_ = *1*/*τ*_*D0*_). *k*_*ET*_ is the ET rate from donor to acceptor and *N* is the ratio of acceptors to donors. In the case of FRET, the interaction between donor and acceptor is characterized by a dipole-dipole interaction resulting in an ET rate that follows a 1/*R*^6^ dependency^1^,^4^,^65^, where *R* is the donor-to-acceptor center-to-center separation distance. The ET rate can be expressed in the following generalized form:


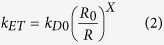


where *R*_0_ is the critical separation distance for which the ET efficiency is 50%. Here, the exponent, *X*, depends on the interaction strength between donors and acceptors as well as their physical nature, their relative size and the separation distance between them. In FRET theory, *X* = 6, since donors and acceptors are considered point dipoles^1^,^4^,^65^. For NSET, as derived by Strouse after modifying Persson’s original surface damping theory^25^,^66^,^67^, the luminescent AuNC is considered to be a fragment of a noble metal surface and the ET rate is expected to follow a 1/*R*^*4*^ dependence (*X* = 4) where *R* is the distance from the AuNC *surface* to the donor.

Our experimental data, however, indicates a weaker dependence of the ET rate on separation distance between the donors and AuNCs, which led us to also consider a further modified version of Persson’s volume damping theory^66^,^67^, referred to as NVET. This considers the luminescent AuNC as a fragment of a noble metal volume with the electron confined to the dimension of the ultra-small AuNC (~1.5 nm), which is significantly smaller than the bulk electron mean free path (20~25 nm)^68^. Here, the effective electron mean free path (*l*_*eff*_ = 1.64 × 0.75 nm (AuNC radius) ≈1.23 nm) and the corrected electron mean free path (*l*_*Cor*_ ~ 2.34 nm)^69^,^70^,^71^ were used for modification of Persson’s theory (see details in the SI). According to NVET, the ET rate is described by Eq. 2 with *X* = 3. The corresponding expressions for each ET model along with their derivation can be found in the SI. Table 2 summarizes the model parameters used to characterize quenching behavior, including the critical distance for each model, *R*_0_, the spectral overlap integral, *J*, the estimated donor-acceptor separation, *R*, (center-to-center distance for FRET, and center-to-metal surface for damping models). We also back-calculated an experimental *R*_0_ (*R*_0*-Exp.*_) from the experimental donor quenching data and donor native lifetime using *R*_0*-Exp.*_ = *(k*_*ET*_/*k*_*D0*_)^*1*/*X*^* × R* for comparison to the theoretically estimated *R*_0_ value postulated by each model. *R* values were calculated based on the approximated length of the ligands or chemical linkers (energy minimized extended forms for the chemical linkers, WLC model for PEG, see SI) and the radius of the NPs from TEM data. The PL spectra of all donors exhibit significant spectral overlap with the AuNC absorption, suggesting that FRET could be a significant quenching mechanism. Calculated spectral overlap integral functions, *J*, ranged from ~2.6 to ~4.1 × 10^15^ M^−1^cm^−1^nm^4^ (Table 2) and the wavelength dependency of *J(λ)* is provided in Fig. S5 for each donor. Similarly, *R*, the donor-acceptor separation for NSET and NVET, are also very favorable for efficient quenching (3.4~9.3 nm) as the calculated NSET *R*_0_ values range from 4.6~7.6 nm while the NVET *R*_0_ values span 7.8~15.2 nm. Given their weaker distance dependency, these are much larger than the FRET *R*_0_ values which ranged from 3.9~5.4 nm.

## Discussion

### Organic Dye Donor-Gold Nanocluster Acceptor Systems: (AuNC)_N_/Cy3 and (AuNC)_N_/Rhodamine-Red

**Figure 6:**
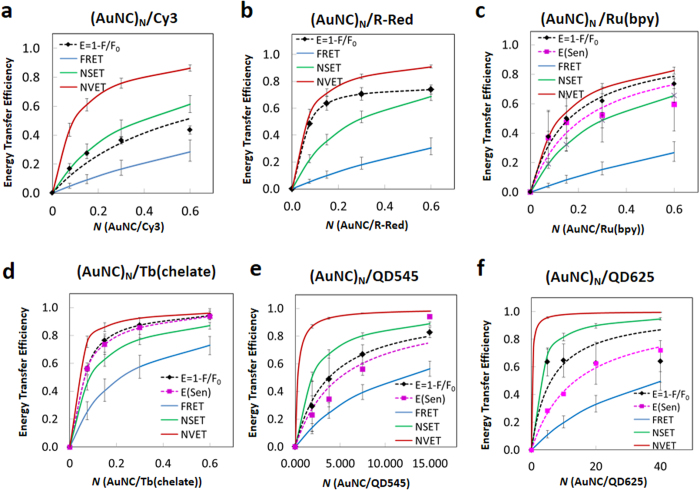
Comparison of ET efficiencies predicted by theoretical models with experimental results. FRET (blue solid line), NSET (green solid line), NVET (red solid line), ET efficiency from donor PL loss (black diamond, fitting based on *k*_*ET*_ in Table 3) and AuNC sensitization (magenta square, line of best fit). (**a**) (AuNC)_N_/Cy3, (**b**) (AuNC)_N_/R-Red, (**c**) (AuNC)_N_/Ru(bpy), (**d**) (AuNC)_N_/Tb(chelate), (**e**) (AuNC)_N_/QD545, and (**f**) (AuNC)_N_/QD625, respectively. Error bars represent the standard deviation of the experimental data, while the error bars on the model predictions represent the extrapolated minima and maxima from the estimated range of donor-acceptor separation distances.

Within the AuNC/Cy3 assemblies, Cy3 PL intensity decreases as *N* increases with no significant changes to its lifetime. R-Red PL undergoes even stronger quenching along with a slight decrease in lifetime *versus N*. The AuNC acceptor in these assemblies exhibits a net decrease in PL intensity, effectively a negative sensitization. Experimental ET efficiency (*E*_*ET*_) from donor steady state fluorescence quenching efficiency (*E*_*Q*_ = *1* − *F*/*F*_*0*_) and acceptor sensitization efficiency, *E*_*Sen*_ (Eq. S4, using the sensitization data where applicable), along with that predicted by the FRET, NSET and NVET models as a function of acceptor number *N* are plotted in Fig. 6. The observed Cy3 quenching was greater than that predicted by FRET theory but was less than the NSET and NVET predictions (Fig. 6a). Dye interactions with proximal AuNPs, even of this small size, *via* NSET are usually characterized by strong quenching of both PL and excited state lifetimes even at small acceptor-donor ratios^25^,^59^,^72^,^73^,^74^,^75^,^76^. Such decreases in Cy3 dye donor lifetimes are not observed under our experimental setup even at these small separation distances. For R-Red, a modest decrease in the average lifetime is seen although this does not decrease beyond ~20% (Fig. 3). Steady state R-Red PL quenching is significantly greater than that predicted by the FRET and NSET models and appears to follow the NVET model more closely (Fig. 6b). Again, we did not observe any sensitization of the AuNC luminescence and similar to the Cy3-AuNC series, a decrease in the AuNC signal was observed. These results and the lack of any AuNC sensitization leads us to conclude that there is no effective ET from the molecular dyes to the AuNCs.

For these organic dyes, the lack of significant changes in lifetime when assembled with AuNCs, in combination with a lack of any AuNC sensitization lead us to rule out the traditional ET mechanisms that have previously been used to describe similar systems. We hypothesize that the dramatic quenching observed in the steady state luminescence of the molecular dyes is the result of efficient intersystem crossing (ISC) from the dye’s first excited singlet state to the triplet state followed by nonradiative relaxation to the ground state. Moreover, the resulting spectra will only reflect dye molecules that are not affected by ISC and thus the PL intensity will change while that of lifetime should not. The ISC rate can be enhanced by strong spin-orbit coupling between the dye and magnetic dipoles on the AuNC surface that are associated with unpaired spins on dangling Au-S bonds, or magnetic properties of the Au core *(vide infra)*^77^,^78^. The slight decrease in the PL intensity of these AuNC complexes may be associated with a small amount of resonant ET from directly excited AuNCs to the molecular dyes. In fact, the very broad AuNC emission band has a small amount of overlap with the absorption spectra of the dyes (See Table S3); this, however, should also not be very significant also matching with our observations.

### Metal Chelate Donor-Gold Nanocluster Acceptor Systems: (AuNC)_N_/Ru(bpy) and (AuNC)_N_/Tb(chelate)

Data from the AuNC/Ru(bpy) assemblies shows the Ru donor undergoing significant PL quenching while the AuNC acceptor sensitization component concomitantly increases as a function of donor number. See Supplementary Fig. S10 for this data plotted as a function of acceptor number (*i.e*., net sensitization per acceptor) for this system along with the QD systems that follow below. The luminescence lifetime of the Ru(bpy) complex decreases modestly with increasing *N*. ET efficiencies, based on steady state luminescence quenching, donor lifetime quenching and AuNC sensitization are shown in Fig. 6c. Efficiency values based on steady state PL and sensitization are in good agreement, but the estimation of ET efficiencies based on donor lifetime quenching is quite low (see Table S4). A similar pattern is seen for the AuNC/Tb(chelate) constructs, although overall changes in the donor lifetime and the acceptor sensitization here are more profound, and the ET efficiencies based on the three types of measurements are in closer agreement. Experimental quenching and sensitization data obtained for the Ru(bpy)- and Tb(chelate)-complexes are compared with the theoretical predictions in Fig. 6c,d. The experimentally determined value of 74% for Ru(bpy) quenching was significantly higher than the ~27% predicted using FRET theory and fell intermediate to the NSET (66%) and NVET (88%) calculations. For the AuNC/Tb(chelate) complexes, steady state donor PL quenching efficiency reached 94% for *N* = 0.6 and this matched with the concomitant acceptor sensitization which reached the same value of 94% for the same ratio. Again, the observed ET efficiency was much higher than the maximum efficiency predicted by FRET but similar to NVET (96%). Additionally, non-conjugate solution phase only control experiments implemented with the metal chelates and the AuNCs at the same concentrations showed almost no ET (data not shown), confirming the critical need for a close proximity between donors and AuNC acceptors for significant ET to occur.

### Quantum Dot Donor-Gold Nanocluster Acceptor Systems: (AuNC)_N_/QD545 and (AuNC)_N_/QD625

Structurally, this configuration represents a true NP-NP system. Within the AuNC/QD545 assemblies, donor PL intensity and lifetime decreased with increasing *N* and were followed by a concomitant AuNC acceptor sensitization. A similar pattern was observed for the QD625 assemblies, with the exception that donor PL did not continue to decrease beyond *N* = 5. Experimental quenching efficiencies for the AuNC/QD assemblies, along with efficiencies predicted by FRET, NSET and NVET are summarized in Fig. 6e,f. The observed donor quenching was again higher than that predicted by FRET but slightly less than the efficiencies predicted by NSET and NVET. Sensitization of the AuNC luminescence within the AuNC/QD545 complex reaches a maximum of 83% for *N* = 15 (see Table S4). The PL quenching efficiency of QD625 reached a maximum for *N* = 5 (64%) and then remained approximately the same with increasing *N*. Maximum AuNC acceptor sensitization by QD625 in this complex was roughly comparable within the error at 72%. The initial quenching efficiency is consistent with the NSET and NVET models and is significantly greater than expected from FRET. Despite the clear and overt AuNC sensitization, the reason for the apparent saturation in the QD625 donor quenching efficiency at *N* = 5 is not clear at this time. ET between QDs and AuNPs has been previously ascribed to an NSET type process^24^,^26^,^59^,^60^,^61^,^62^,^63^,^64^.

Ensemble measurements of QD lifetimes are typically described by an ensemble average value (*τ*_avg_) composed of multiple exponential decays which have both short and long-lived components^79^,^80^. ET from QDs to AuNC acceptors should also be influenced by the multicomponent decay processes. Examination of the relative quenching of these components within the AuNC/QD assembly shows the long-lived component being significantly more quenched than the short-lived component, see Table S2 and Fig. S9. One possible explanation for this observation is that the long-lived components represent ensemble QDs with higher *Φ’*s than those associated with the short-lived components^3^. Therefore competition between nonradiative decay and ET favors the ET process for the longer-lived components. Overall, there is clear evidence of AuNC sensitization resulting from interaction with QDs and the quenching of the QDs is stronger in magnitude than that predicted by FRET.

Summarizing, for all three donor types, FRET calculations greatly underestimate *E*_*ET*_, while the NSET/NVET model predictions were much closer. Although both the NSET and NVET models provided qualitatively better fits than the FRET model, especially for the Ru(bpy) and Tb(chelate) donor assemblies, they still do not fully reproduce the observed behavior. To provide further insight into the ET mechanism(s), in Table 3 we also estimated the average ET rates (*k*_*ET*_) for the three models from donor quenching and acceptor sensitization efficiency using Eq. 2 in conjunction with the theoretical critical separation (*R*_0_) and our experimentally-derived data for the distance *R*_0*-Exp*_ (Table 2). Using the reciprocal of the averaged native donor decay time (*1*/*τ*_*D0*_), we estimated the donor decay rate (*k*_*D0*_) and extracted the ratio of experimental ET rate to donor decay rate, *k*_*ET*_/*k*_*D0*_, shown in Table 3 (details in SI). This shows that *k*_*ET*_/*k*_*D0*_ directly affects the ET efficiency, *E*_*ET*_ (Eq. 1), with the larger *k*_*ET*_/*k*_*D0*_ values correlating with the “efficient” ET configurations.

### Unique Electronic Characteristics of AuNC Acceptors

Given the lack of an across-the-board strong correlation between predicted and observed ET efficiencies for all the different donor materials and any of the model treatments in Fig. 6, we look to other factors associated with the AuNCs which could contribute to or otherwise influence such interactions. These are discussed from a predominantly theoretical perspective and begin with the unique electronic nature of the AuNCs themselves before examining the role of magnetism.

The electronic structure of the luminescent AuNCs is significantly different from that of traditional molecular dye acceptors. AuNCs have a very high density of excited states, *ρ(E)*, and an associated fast dephasing time, *τ*_*cA*_ ~ 10 fs, based on the ~410 meV spectral bandwidth of the AuNC PL^81^. These factors may also contribute significantly to the high ET efficiency observed within some of the AuNC-acceptor constructs. The dephasing time refers to the amount of time, following coherent ET, required for the excited electronic states of the donor-acceptor pair to become completely incoherent. At this point, the probability of (resonant) back-transfer is exceptionally low. The mechanisms involved in the spectral broadening and rapid dephasing in AuNCs are described in detail by Wen *et al*.^82^.

Here, we define the resonant ET rate from a donor to a AuNC acceptor as *k*_*ET*_ = (2π/*ℏ*)(*V*_*DA*_)^2^*ρ*(*E*), where the matrix element *V*_*DA*_ describes the coupling between the donor and acceptor excited states. The density of excited states in these types of small-sized AuNCs can be estimated as *ρ(E)* ≈ *Mτ*_*cA*_/*ℏ*, where *M* is the number of acceptor excited states, *ℏ* is the reduced Planck constant and *ℏ*/*τ*_*cA*_ is the line-width of the acceptor resonance. The donor-AuNC-acceptor ET rate can then be estimated as:


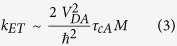


where *M* ≫ 1. This explains the efficient ET to the AuNC acceptors that was observed from the metal ion complexes and QDs. As *M* becomes larger, *k*_*ET*_ will also become larger in proportion to *M*. Thus, compared to an acceptor having a smaller *M* value, the AuNC is expected to have a more efficient ET rate from the same donor.

We next look to the excitation dynamics in an ensemble of optically active donors and AuNC acceptors which are coupled *via* an ET process. The time dependence of the excited donor and acceptor populations, *n*_*D*_ and *n*_*A*_, respectively, under steady state (continuous wave) excitation conditions can be described by the kinetic equations:


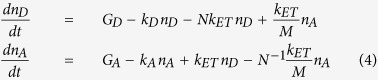


where *G*_*D*_ and *G*_*A*_ are the probabilities of donor and acceptor excitation, *k*_*D*_ and *k*_*A*_ are the decay rates of excited donor and acceptor, *N* is the ratio of acceptor to donor in the ensemble, and *k*_*ET*_ is the ET rate. The steady state solutions of Eq. 4 are


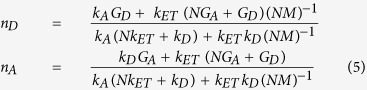


From Eq. 5 it is apparent that, in the absence of ET (*k*_*ET*_ = 0), the donor and acceptor populations are determined by the two standard independent expressions: *n*_*D*_* *=* G*_*D*_/*k*_*D*_ and *n*_*A*_ = *G*_*A*_/*k*_*A*_. Assuming the *M* ≫ *1* condition is satisfied and transitions from the acceptors to donors are suppressed, transparent expressions for the donor and acceptor populations become:


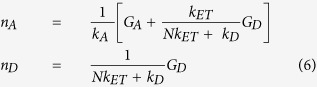


Eq. 6 confirms that ET between donor and acceptor increases the population of the acceptor states, *n*_*A*_, and reduces the population of donor states, *n*_*D*_, conseque*n*tly increasing the PL intensity of the acceptors while suppressing donor PL. The PL intensities of donor and acceptor, *n*_*D*_*k*_*D*_ and *n*_*A*_*k*_*A*_*N*, respectively, depend on the product of the acceptor-to-donor ratio, *N*, and the ET rate *k*_*ET*_. Sensitization of acceptors as introduced by Dexter is described as *N*(*n*_*A*_*k*_*A*_* − G*_*A*_)^83^. Using Eq. 6, we obtain the sensitization *N*(*n*_*A*_*k*_*A*_* − G*_*A*_) = *E*_*ET*_ *G*_*D*_ with the ET efficiency given by Eq. 1. See SI for some additional calculations and discussion of the coupling parameter between donor and acceptor excited states, *V*_*DA*_, based on experimental data.

### AuNC Magnetism and Its Effect on Intersystem Crossing

Data from the Cy3 and R-Red organic dye-AuNC assemblies show very effective quenching of both donors and a slight quenching of the acceptor PL. Suppression of PL from Cy3 and R-Red molecules in the presence of heavy atoms has been well documented^84^ and was connected with the activation of a thermalization channel which effectively transfers excitation to the lowest optically passive state where nonradiative recombination completely quenches the PL. In these molecules, the emissive states are singlets and the dark states are triplets and transitions between them require a spin flip process, which normally has a lower probability than emission from the singlet state^2^,^3^. Similarly, the efficient quenching of PL from the organic dye donors in the presence of AuNCs may well be connected with fast spin-flip transitions facilitated by dangling bonds (unpaired spins) at the surface of the AuNCs. Indeed, magnetic properties of unpaired spins were recently proposed for QDs^85^,^86^,^87^,^88^ and AuNCs^77^.

The quenching of both donor molecules and the AuNC acceptor can be described using the system of Eq. 4 and 5, assuming that the relaxation rate of the donor, *k*_*D*_, is increased significantly and this relaxation becomes mainly nonradiative. The populations of the emissive states in the donor and acceptor are still governed by Eq. 4, wherein the donor decay rate is the sum of the radiative and nonradiative components *k*_*D*_* *=* k*_*r*_* *+* k*_*nr*_. Although the solutions for *n*_*D*_ and *n*_*A*_ can be written in the same form as in Eq. 5, the luminescence intensities are different. Here, fast nonradiative donor decay can completely predominate over the ET from donor to acceptor when *k*_*nr*_ ≫ *k*_*ET*_. The PL intensity of the donor, which is proportional to *I*_*D*_* *=* k*_*r*_ *n*_*D*_ and can be rewritten using Eq. 6 as *I*_*D*_ = [*k*_*r*_/(*N k*_*ET*_ + *k*_*D*_)]*G*_*D*_ ≈ (*k*_*r*_/*k*_*nr*_) *G*_*D*_ ≪ *G*_*D*_. Resonant energy transfer from the AuNCs to the dyes, for example, is described by 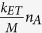 in Eq. 4. This assumes: that (*i*) sufficient spectral overlap exists for this condition, in reality the actual values are negligible and *circa* 2 orders of magnitude less (SI Table S3) than those for each donor to the AuNC as acceptor (Table 2); and (*ii*) *k*_*D*_ ≫ *k*_*ET*_ in Eq. 5, then we can obtain the acceptor population:


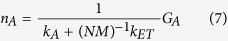


In this case, the sensitization is negative in agreement with Fig. 3a,d and Table S4 for Cy3 and R-Red based on:


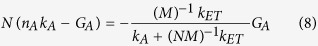


Here, one can see that ET depletes the acceptor population and consequently it decreases the PL intensity which is proportional to *n*_*A*_*k*_*A*_. The non-radiative channels also lead to an unaccounted loss of excitation when the donor quenching *G*_*D*_ − *k*_*r*_*n*_*D*_ exceeds the acceptor sensitization *N*(*n*_*A*_*k*_*A*_ − *G*_*A*_).

To verify the possibility of paramagnetic species on the surface of the AuNCs that would contribute to fast spin-flip transitions, we performed SQUID (superconducting quantum interference device) magnetic measurements using a Quantum Design Magnetic Properties Measurement System^89^. Measurements of magnetic moment *vs*. temperature (*T*) were analyzed using a modified Curie-Weiss equation to derive the magnetic susceptibility, see SI^85^, and confirmed the existence of AuNC paramagnetic centers. Supplementary Fig. S11 shows the experimental results and fitting parameters in terms of absolute number of paramagnetic centers assuming they behave as isolated spin 1/2 entities since we were unable to determine an accurate density value for the clusters. The small temperature (*θ*) value indicates minimal electron-electron exchange interactions typical of isolated paramagnetic centers. The quality of fit to the nearly *1*/*T* behavior of susceptibility also indicates the presence of localized moments in the compound. The total number of spins (~2 × 10^17^) measured along with the total weight of AuNC (~6 mg) allows us to roughly estimate the average number of surface dangling bond spins at ~2 spins per AuNC (details in SI).

### Differences in Observed Donor Steady-State *versus* Lifetime Quenching

Additional evidence for AuNC acceptors influencing organic dye donors may be found in the difference in quenching efficiency estimates when using either steady state or lifetime measurements. The quenching efficiencies based on steady state experiments (*E*_*ET*_ = *1* − *F*_*D*_/*F*_*D0*_) always appear larger than those based on time decay experiments (*E*_*ET*_ = *1* − *τ*_*D*_/*τ*_*D0*_ used for the traditional ET calculations here). The PL intensity of donor, *F*_*D*_, is proportional to (1/*τ*_*Dr*_)/(1/*τ*_*D*_), where *τ*_*Dr*_ and*τ*_*D*_ are the radiative and total decay time of a donor, respectively. The total decay rate, 1/*τ*_*D*_, in the presence of the AuNCs includes the ET rate. As a result, the ratio of donor PL intensity in the presence of AuNCs, *F*_*D*_, and in their absence, *F*_*D0*_, can be written as *F*_*D*_/*F*_*D0*_ = (*τ*_*D*_/*τ*_*D0*_)(*τ*_*Dr0*_/*τ*_*Dr*_) where *τ*_*Dr0*_ and *τ*_*D0*_ are the radiative and total decay times of the donor in the absence of the AuNCs. One can see that steady state measurements of the ET efficiency, 1 − *F*_*D*_/*F*_*D0*_, contains the additional factor *τ*_*Dr0*_/*τ*_*Dr*_, which is usually “1” for traditional ET calculations; assuming the radiative decay rate of donor does not change with/without acceptor. This can explain the difference if the radiative decay time, *τ*_*Dr*_, becomes longer in the presence of AuNCs (*τ*_*Dr0*_/*τ*_*Dr*_ < 1), and *1 − F*_*D*_/*F*_*D0*_ = 1 − (*τ*_*D*_/*τ*_*D0*_)(*τ*_*Dr0*_/*τ*_*Dr*_) is larger than the traditional lifetime based *E*_*ET*_ calculation, *1 − τ*_*D*_/*τ*_*D0*_. Supporting the notion of variation in radiative/nonradiative decay rates by gold nanoparticulates, an increase in Lissamine and Cy5 donor dye decay times in the presence of AuNCs was observed in refs 90 and 91 while, in contrast, a reduction in the donor radiative decay time induced by an AuNP was also recently reported^92^.

## Conclusions

FRET is characterized as a classical dipole-dipole coupling where the ET rate is almost completely dependent on the donor and acceptor decay rates^2^,^3^,^4^. This situation is generally satisfied when utilizing small molecular fluorophores as they display discrete, well-defined electronic states. Although, the QD’s non-trivial size in conjunction with their confined electronic structure and the amorphous nature of their dipoles make QDs more complex, it is generally accepted that they, too, engage in a similar type of FRET coupling^4^,^13^. It is also generally accepted that the PL quenching of both molecular fluorophore- and QD donors by non-emissive AuNP acceptors is *not* due to a classical FRET process. Analogous to the different types of ET processes put forth to describe AuNC sensitization in the Introduction, a similar set of diverse processes has also been suggested to govern ET quenching by AuNPs. These include FRET^90^,^93^,^94^,^95^,^96^, NSET^1^,^59^,^72^,^97^,^98^, or, alternatively, neither of these processes^99^,^100^,^101^,^102^. It has also been proposed that donor quenching efficiency may be dependent on AuNP acceptor size or surface properties^90^,^94^,^95^,^103^,^104^,^105^,^106^. Given this lack of generalized agreement, it should not be surprising that ET and sensitization of AuNCs, most simplistically just a smaller AuNP with PL properties, would also be assigned to a mix of both FRET and NSET processes and also not definitively match with either of these models.

Here, we confirm that luminescent AuNCs do indeed function as efficient ET acceptors when paired with appropriate donors displaying the requisite electronic characteristics, *i.e*. luminescent lowest energy excited states, such as the Ru and Tb metal ion complexes along with QDs. AuNC sensitization by the Tb(chelate) and QDs may indeed represent the first reports of this phenomena with these particular donor materials. We also note that many of the observed photophysical changes in donor to AuNC acceptor properties during ET are quite similar to those expected as “Hallmarks” or defining characteristics of classical FRET and this can serve to confuse the interpretation. For example, steady state donor quenching was matched with a concomitant increase in AuNC acceptor sensitization and, similarly, donor lifetime decreases with increases in that of the paired AuNC acceptor. The magnitude of these photophysical changes track to some extent directly with the number of acceptors or donors in the experimental configuration towards a plateau. Moreover, the observed ET efficiency is also dependent upon the requisite spectral overlap being present along with close donor-acceptor proximity; this strongly suggests the need for resonance between donor-acceptor dipoles. The experimentally determined ET efficiencies were rigorously compared to predicted efficiencies based on the FRET, NSET and NVET models all of which have a strong distance-dependent coupling. In all cases, the FRET model dramatically underestimated the efficiencies while closer approximations to the experimental data were provided by modifying Persson’s surface and volume damping theory; the latter is not based on a traditional FRET coupling.

In contrast to the above, the Cy3 and R-Red organic dyes do not appear to manifest any notable role as ET donor to the AuNCs. The slight AuNC quenching in the presence of dyes may be due to ET from directly-excited AuNCs to the dyes. However, the small amount of underlying spectral overlap (SI Table S3), suggests that this is not a dominant or primary mechanism. This type of ET would be associated with weak or incoherent coupling similar to Förster energy transfer^107^. The possibility also exists for some type of non-descript fluorescence quenching induced by the metallic nature of the proximal NPs along with that arising from a “heavy atom effect”^84^,^108^,^109^. Within our configurations, the lack of AuNC sensitization by the dyes is partially attributed to intersystem crossing between the lowest excited singlet states and the triplet states of the dyes. The paramagnetism observed in the AuNCs, which arises from the presence of dangling bonds, supports this possibility. Although our lifetime capabilities do not extend into the femtosecond regime, we do see a decrease in the R-Red lifetime when coupled to AuNCs which would be expected for such a component. Supporting our conjecture of the need to pair AuNCs with an appropriate donor, QDs do not decay from triplet states at room temperature and metal complexes such as the Tb(chelate) and Ru(bpy) access symmetry and spin forbidden states when excited^51^,^110^,^111^.

Cumulative contributions from their electronic structure, exceptionally high density of excited states, fast dephasing time, and even magnetism all contribute to making AuNCs a “non-classical” material when it comes to ET as these factors must now all be considered and even accounted for. Additionally, in the role of an acceptor the net result of several AuNC properties (broad absorption spectrum extending from the UV to the near-IR, relatively large, wavelength-dependent optical cross-sections and PL lifetimes approaching 1 μs) mean that, in addition to ET from donors, there will also be a direct acceptor excitation component which can significantly complicate experimental formats. Using a theoretical analysis extended from our results, we suggest that AuNC properties, including especially the rapid dephasing time (~10 fs), play an important role in efficient ET by effectively preventing resonant back-transfer to the donor which has a relatively long lifetime compared to this. This situation is different from a classical Förster mechanism (*i.e*., incoherent) and has been referred to as coherent ET *via* strong coupling between the donor and acceptor^107^. As our estimated separation distances (4.5~5 nm of center-to-center distance) between the dyes and AuNC is longer than that expected for an efficient electron transfer mechanism (especially in buffer), we do not evaluate it as a quenching mechanism although we cannot completely exclude it. However, electron transfer would manifest as a decrease in both donor and acceptor PL and their lifetimes which is something we do not observe in the metal complex and QD-AuNC systems. The fact that the metal complex and QD systems show ratiometric changes to their lifetimes also strongly argues against any static quenching mechanism^2^. The presence of an isosbestic point around which the AuNC acceptor PL increases in a manner that directly tracks with monotonic Ru(bpy) donor PL decreases (see Fig. 4A) also argues against a static quenching mechanism. Moreover, the experimental concentrations utilized here are far lower than those typically used to achieve static quenching^2^. There is also an isosbestic point within the AuNC/Tb(chelate) data (Fig. 6d), *albeit* of much smaller magnitude. Indeed, such data is quite analogous to the dynamic process originally observed for sensitization of near-IR emitting 1.8 nm diameter tiopronin-protected AuNCs with (Ru(bpy)_3_)^2+^ donors^23^. A similar donor quenching/acceptor sensitization around a central isosbestic point was also reported with blue-emitting silsesquioxane polyhedral oligomeric donors sensitizing BSA-encapsulated 700 nm emitting AuNC acceptors^30^. We also considered the possibility of contributions from a color conversion mechanism, however, estimates based on experimental conditions including concentration using the calculations described in ref. 112. suggest a negligible effect with <1% predicted as a maximum contribution (data not shown).

Critically, of all the processes considered, FRET appears to provide the poorest match with the experimental data. Although qualitatively better, the metal dampening models also fail to fully account for the observed trends. Thus, we suggest that, at the least, FRET formalism should *not* be used to analyze ET in these types of AuNC acceptor experimental configurations. Similarly, if the metal dampening models are used with AuNC acceptors, they should be carefully implemented with the knowledge that they, too, are still incomplete descriptions. As an interim remedy, we found that the use of Equation 6 to fit these types of experimental data is quite reasonable as it combines relative donor and acceptor ratios, their photophysical changes, and ET rates with *no* explicit distance dependence factored in. We again stipulate that these are working hypotheses from the interpretation of the experimental data, meaning that our interpretation could have errors; they are rather meant to suggest a useful approach until such time as more data and a fuller understanding is brought forth. An alternative approach is to separate FRET from NSET processes in assemblies that incorporate both multiple dyes and small AuNPs^113^. Despite these caveats, AuNC sensitization by an appropriate donor may still prove to be quite useful for an “on/off” type of biosensor, especially if near-IR acceptor emission is required such as in deep tissues for example. Lastly, it is also important to consider that FRET itself may not be as rigid as initially postulated, but rather more nuanced and this is being repeatedly proven with accumulating examples within ever more sophisticated configurations^57^,^58^,^114^.

In terms of future avenues of research, experiments that systematically and precisely vary the donor-AuNC acceptor separation may help to better understand the underlying photophysical nature of the interaction(s). As the electronic properties of luminescent AuNCs are elucidated, there will also be a need for more physical evidence supporting coupling *via* an interaction of a donor’s transitional dipole with electron fluctuations in the AuNC along with the role of AuNC magnetism. AuNC synthesis also continues to rapidly mature and attain far higher *Φ*^115^, which is, in turn, contributing to making them more attractive ET acceptors. Clearly, a full understanding of their function in this role will certainly help promote their wider application.

## Additional Information

**How to cite this article**: Oh, E. *et al*. Energy Transfer Sensitization of Luminescent Gold Nanoclusters: More than Just the Classical Förster Mechanism. *Sci. Rep.* **6**, 35538; doi: 10.1038/srep35538 (2016).

## Supplementary Material

Supplementary Information

## Figures and Tables

**Table 1:** AuNC Acceptor and Donor Optical Properties.

	*Φ*	Abs(max, nm)	Em(max, nm)	*ɛ* (M^−1^cm^−1^)	*τ* (s)	*k*_*r*0_ (s^−1^)	*k*_*nr0*_(s^−1^)
AuNC	0.06	—	820^*^	4.5 × 10^4^ (520 nm)	7.5 × 10^−7^	8.0 × 10^4^	1.3 × 10^6^
Cy3	0.09	550	570	1.5 × 10^5^	5.7 × 10^−10^	1.6 × 10^8^	1.6 × 10^9^
R-Red	0.20	570	580	1.3 × 10^5^	2.8 × 10^−9^	7.2 × 10^7^	2.9 × 10^8^
Ru(bpy)	0.07	445	615	1.8 × 10^4^	3.9 × 10^−7^	1.8 × 10^5^	2.4 × 10^6^
Tb(chelate)	0.30	343	545	1.3 × 10^4^	9.1 × 10^−4^	3.3 × 10^2^	7.7 × 10^2^
QD545	0.16	533	545	1.6 × 10^5^	7.3 × 10^−9^	2.2 × 10^7^	1.2 × 10^8^
QD625	0.50	611	625	5.0 × 10^5^	6.1 × 10^−8^	8.2 × 10^6^	8.2 × 10^6^

*Φ* = quantum yield, Abs(max) = maximum absorption peak/QD first absorption band, Em(max)* *=* *maximum emission wavelength, *ɛ *=* *extinction coefficient, *τ *=* *amplitude weighted averaged lifetime, *k*_*r0*_ = radiative decay rate, *k*_*nr0*_ = nonradiative decay rate. ^*^FWHM ~220 nm.

**Table 2:** Relevant Parameters for FRET and Metal Damping Energy Transfer Models.

Donor	*R* (Å)^a^ (range)^b^	*J* (M^−1^cm^−1^nm^4^)	Theoretical *R*_0_ (Å) FRET/NSET/NVET	Experimental *R*_0*-Exp*_ (Å)^c^ FRET/NSET/NVET
Cy3	45 (42–47)	3.1 × 10^15^	42/47/81	49/43/45
R-Red	50 (47~52)	2.9 × 10^15^	47/58/107	73/75/91
Ru(bpy)	42 (40~44)	2.6 × 10^15^	39/46/78	57/54/63
Tb(chelate)	42 (39~44)	4.1 × 10^15^	53/62/117	68/71/91
QD545	70 (67–72)	3.3 × 10^15^	46/53/95	56/45/40
QD625	101 (96~105)	2.7 × 10^15^	54/76/152	74/59/51

^a^Center-to-center distance between acceptor and donor. Ligand length and dye size were calculated based on crystal structure and energy minimization (ChemDraw, PerkinElmer, Inc.). PEG size was calculated using a Worm-Like-Chain model. Sizes utilized here are Cy3: 9~12 Å, R-Red: 14~16 Å, Ru(bpy): 6~9 Å, Tb(Chelate): 6~8 Å, MUA: ~15 Å, CL1: ~20 Å, TA-PEG-NH_2_: 26~29 Å.

^b^Parenthetical values represents uncertainty of each calculation based on standard deviation of the sum of independent variables (size of ligand, size of NPs); SD(X + Y) = 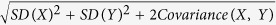. These are used as minimal/maximal boundaries for each size estimate.

^c^Critical separation distance for FRET is the donor center-to-acceptor center separation, for NSET/NVET it is the donor center-to-acceptor surface separation. Experimental *R*_0_ (*R*_0*-Exp*_) calculated from experimental data using *(k*_*ET*_/*k*_*D0*_)^*1*/*X*^* × R* with X = 6, 4, 3 for FRET, NSET and NVET, respectively. These can be different from theoretical values when calculated directly using each energy transfer model.

**Table 3:** Energy Transfer Rates.

Donor	Energy Transfer Rate, *k*_*ET*_ (s^−1^)				
	Donor Quenching^a^	AuNC Sensitization^b^	FRET	NSET	NVET
Ru(bpy)	1.6 × 10^7^ (6.2)	1.2 × 10^7^ (4.5)	1.6 × 10^6^	8.2 × 10^6^	3.0 × 10^7^
Tb(chelate)	2.1 × 10^4^ (19.2)	2.0 × 10^4^ (18.3)	5.0 × 10^3^	1.2 × 10^4^	4.5 × 10^4^
QD545	3.7 × 10^7^ (0.3)	2.8 × 10^7^ (0.2)	1.2 × 10^7^	7.5 × 10^7^	5.0 × 10^8^
QD625	2.7 × 10^6^ (0.2)	1.2 × 10^6^ (0.1)	4.0 × 10^5^	7.3 × 10^6^	7.2 × 10^7^

^a,b^Parenthetical values are the ratio of the energy transfer rate (*k*_*ET*_ experimental) to donor decay rate, *k*_*ET*_/*k*_*D0*_.
