# Supplementary material for: Energy Transfer Sensitization of Luminescent Gold Nanoclusters: More than Just the Classical Förster Mechanism
Source: Sci Rep. 2016 Oct 24;6:35538. doi: 10.1038/srep35538 (PMC5075882; doi:10.1038/srep35538)
Supplement: Supplementary Information [file srep35538-s1.pdf]

Supplementary Information

Energy Transfer Sensitization of Luminescent Gold Nanoclusters: More Than Just The Classical Förster Mechanism

Eunkeu Oh[1,2,*], Alan L. Huston[2], Andrew Shabaev[3], Alexander Efros[3], Marc Currie[2], Kimihiro Susumu[1,2], Konrad Bussmann[4], Ramasis Goswami[4], Fredrik K. Fatemi[2], and Igor L. Medintz[5,*]

**Contents**	**Page**
**Supplementary Materials and Methods**	**S-3**
**Chemicals**	**S-3**
**Quantum Dots**	**S-3**
**Cap Exchange of Quantum Dots**	**S-3**
**Gold Nanoclusters**	**S-3**
**Conjugation of Donors to AuNC Acceptors**	**S-4**
**Electron Microscopy**	**S-5**
**Dynamic Light Scattering**	**S-5**
**Steady-State Optical Characterization**	**S-5**
**Fluorescence Lifetime Measurements**	**S-6**
**SQUID**	**S-6**
**Quantum Yield Measurements**	**S-7**
**Determination of Energy Transfer Efficiency**	**S-8**
**I. Donor Luminescence Quenching**	**S-8**
**II. Acceptor Luminescence Sensitization**	**S-8**
**Energy Transfer Analysis**	**S-9**
**I. Förster Resonance Energy Transfer**	**S-9**
**II. Surface/Volume Damping**	**S-10**
**Volume Damping Theory Modification**	**S-12**
**Decay Rate Constant Calculation**	**S-14**
**Energy Transfer Decay Rate During Donor Quenching**	**S-14**
**Coupling Parameter Calculation**	**S-15**
**Worm-Like-Chain Model**	**S-16**
**Increased Energy Transfer Probability From Tb(chelate)**	**S-28**
**Calculation of Spin Number per AuNC**	**S-30**
**Approach 1**	**S-30**
**Approach 2**	**S-31**
**Supporting References**	**S-31**

**Contents**	**Page**

**Table S1. TEM, DLS, Zeta-Potential**	**S-21**
**Table S2. Summary of Donor and Acceptor Lifetimes**	**S-24**
**Table S3. AuNC Acceptor and Donor Optical Properties**	**S-26**
**Table S4. Experimental and Modeled Energy Transfer Efficiencies**	**S-27**
**Figure S1. Chemical structures of materials used.**	**S-17**
**Figure S2. Optical properties of acceptor/donor materials.**	**S-18**
**Figure S3. Physical characteristics of AuNC.**	**S-19**
**Figure S4. The optical characteristics of AuNC.**	**S-20**
**Figure S5. The spectral overlap.**	**S-22**
**Figure S6. Emission profile and quenching of the Tb(chelate)**	**S-23**
**Figure S7. Fluorescence image of $(AuNC)_N$-QD545**	**S-23**
**Figure S8. The spectrum of the 532 nm excitation pulse**	**S-23**
**Figure S9. Changes in the biexponential lifetime components**	**S-25**
**Figure S10. The net sensitization signal enhancement rate**	**S-29**
**Figure S11. SQUID measurement from AuNC**	**S-30**

Supplementary Materials and Methods

Chemicals: *N*-hydroxysuccinimide-Cy3 dye (Cy3-NHS) was purchased from GE Healthcare. Bis(2,2′-bipyridine)-(5-isothiocyanato-phenanthroline)ruthenium bis(hexafluorophosphate) (Ru-NCS) was purchased from Sigma. Rhodamine Red™-X (R-Red-NHS) and LanthaScreen™ Amine Reactive Tb(chelate)-NCS, Triethylenetetramine-*N*,*N*,*N*',*N*',*N*'',*N*''-hexaacetic acid isothiocyanate-Carbostyril 124, were purchased from Invitrogen. All of the other chemicals (including solvents) were purchased from Sigma Aldrich or Acros Organics and used as received.

Quantum Dots: CdSe-CdS-CdZnS-ZnS core-multishell QDs (QD545) were synthesized following previously published procedures with some modifications.[1] QD synthesis was carried out under dry nitrogen, and all air-sensitive solids were handled in a M. Braun Labmaster 130 glovebox (Stratham, NH). Custom Qdot®625 nm-emitting ITK® (QD625) was obtained from Life Technologies (Eugene, OR).

Cap Exchange of Quantum Dots: The QDs were cap-exchanged with relatively short-length ligands to provide a negatively charged surface which would allow for electrostatic interaction with the positively charged amines on the AuNCs and bring them into a close-surrounding proximity. QD545 was cap-exchanged using mercaptoundecanoic acid (MUA) while QD625 was cap-exchanged with a carboxy-functionalized zwitterionic compact ligand (CL1) synthesized as described.[1] These short ligands position the QD donors at distances that should allow for efficient energy transfer to the AuNCs.

TA-PEG Ligands**:** Poly(ethylene glycol)-modified thioctic acid ligands with a terminal amine (TA-PEG-NH_2) were synthesized, purified, and characterized as previously described in ref 2.[2] The average molecular weight of PEG was ≈600 ± 30, consisting of approximately 12 ethylene oxide repeat units, m ≈ 12), see Scheme 1.

Gold Nanoclusters: AuNCs were synthesized following published procedures with minor modifications.[3-5] Briefly, 156 µl (1.56×10^{-5} mol) of 100 mM tetrachloroauric (III) acid ($HAuCl_4\cdot3H_2O$) aqueous stock solution and 586 µl (7.8×10^{-5} mol) of 133 mM of TA-PEG-NH_2 aqueous stock solution were mixed with 50 µl of deionized water. The mixture was then stirred at room temperature for 0.5 h. 312 µl (3.12×10^{-4} mol) of 1 M sodium borohydride ($NaBH_4$) stock solution, in deionized water, was added in two aliquots of 156 µl with vigorous stirring.

Following addition of the reducing agent the color of the reaction mixture immediately changed from clear to light brown. This mixture was stirred for at least 3 h while the reaction gradually progressed to completion. For aging and ripening, the clusters were stored for an additional two days at room temperature without stirring. The dispersion was then purified by removal of the free ligands with three cycles of centrifugation using a membrane filtration device (10 K molecular weight cut-off, Millipore Corporation). AuNC dispersions were characterized using UV-Vis absorption spectroscopy and TEM as described below.

Conjugation of Donors to AuNC Acceptors*:* We attached amine-reactive Cy3-NHS and (R-Red-NHS) to the amine-terminated AuNCs through amide bond formation. Similarly, isothiocyanate-containing amine-reactive Ru(bpy) and Tb(chelate) were used to form isothiourea bonds with the amine-terminated AuNCs. The CL1- and MUA-functionalized QDs have terminal carboxyl groups that facilitate self-assembly with the amine-functionalized AuNCs via electrostatic interactions. This is driven by the low pKa of the alkyl carboxylic acids (~4.8) and the high pKa of the primary amine group (∼10),[6,7] which means both will be mostly ionized at around the neutral pH of water. Zeta potential was measured to confirm the ionization of carboxylated QD (~ -42 mV) and amine-terminated AuNC (~13 mV) before self-assembly, see Table S1 in Supporting Information (SI). 40 μM stock solutions of the dyes were prepared using 10% DMSO in water. 4 μM stock solutions of QD545 and QD625 were prepared in pure water. AuNC-Cy3/R-Red/Ru(bpy)/Tb(chelate) acceptor/donor conjugates were prepared using Configuration 1 (Scheme 1). Different concentrations of the AuNC/conjugates were assembled by fixing the amount of Cy3-NHS, R-Red-NHS, Ru(bpy)-NCS and Tb(chelate)-NCS at 5.3 μM and altering the relative AuNC stoichiometric concentration accordingly. This resulted in acceptor-to-donor ratios (N) of 0.08, 0.15, 0.3 and 0.6 (each AuNC surrounded by an average of ~13.3, 6.7, 3.3 and 1.7 donors). AuNC/QD conjugates, based on Configuration 2 (Scheme 1), were prepared as follows: 200 nM solution of QD545 was mixed with appropriate concentrations of AuNC solutions to produce AuNC/QD545 ratios of 2, 4, 8 and 15. Similarly, an 80 nM solution of QD625 was mixed with AuNC solutions to yield N of 5, 10, 20 and 40. After mixing, reaction solutions were kept at room temperature for 2 h without stirring prior to performing any experiments. We note that analogous assembly approaches have been utilized with similar materials previously.[8,9]

Electron Microscopy: Structural characterization of the AuNCs and QDs was carried out using a JEOL 2200-FX analytical high-resolution transmission electron microscope with a 200 kV accelerating voltage. Samples for transmission electron microscopy (TEM) and scanning transmission electron microscopy (STEM) were prepared by spreading a drop of the AuNC dispersion (or AuNC/QDs dispersion) onto a holey carbon film on a fine mesh Cu grid (400 mesh) and letting it dry. The same technique was used for the QDs except an Au grid was used instead of the Cu grid. The concentration of NPs was ~10 μM. The average particle size and standard deviation was determined using a Gatan Digital Micrograph, by averaging of at least ~100 NPs.[3,10]

Dynamic Light Scattering: Dynamic light scattering (DLS) measurements were carried out using a CGS-3 goniometer system equipped with 633 nm HeNe laser illumination and a single-photon counting avalanche photodiode for signal detection. The autocorrelation function was performed by an ALV-5000/EPP photon correlator (ALV, Langen, Germany) and analyzed using Dispersion Technology Software (DTS, Malvern Instruments). AuNC solutions were prefiltered through 0.25 µm syringe filters (Millipore) prior to DLS measurements to remove dust or impurities in the sample. Sample temperature was maintained at 20°C. For each sample, the autocorrelation function was the average of three runs of 10 s each and then repeated at different scattering angles (within 70° and 140°). CONTIN analysis was then used to extract intensity, volume, and number versus hydrodynamic size profiles for the dispersions studied.[3,11]

Steady-State Optical Characterization: Electronic absorption spectra were recorded using an HP 8453 diode array spectrophotometer (Agilent Technologies, Santa Clara, CA). Fluorescence spectra were collected using a Spex Fluorolog-3 spectrophotometer (JobinYvon Inc, Edison, NJ) equipped with a red-sensitive R2658 Hamamatsu photomultiplier tube (PMT) for wavelength-scanned spectra, and a liquid nitrogen-cooled CCD array for collection of dispersed spectra. Sample excitation was accomplished using a monochromatized xenon lamp. The fluorescence spectra were corrected using the spectral output of a NIST-certified calibrated light source. The excitation wavelength was 470 nm for the steady-state fluorescence measurement except for the 350 nm used with the Tb(chelate).

Fluorescence Lifetime Measurements: We used two different laser systems (355 nm and 532 nm) as excitation sources for lifetime measurements. The fluorescence lifetimes of all AuNC

complexes, except the Tb(chelate)-AuNC and Ru(bpy)-AuNC systems, were measured using an amplified Ti:sapphire laser (Coherent RegA 9040) that was used to pump an optical parametric amplifier (Coherent OPA 9400) to produce 8 nm bandwidth optical pulses at 532 nm with a <70 fs pulse width at a repetition rate of 250 kHz and with an average power of ≈2 mW. The output was passed through a 50-nm bandpass filter to remove any residual 800 nm pump and white-light signals. The 532-nm pulsed light was collimated to ≈2 mm diameter and was directed through a 1 cm path length cuvette containing the sample solution. The luminescence lifetimes of the donors and the AuNC complexes were measured by coupling the signal light into a fiber array placed at 90° to the 532-nm excitation direction. The output of the fiber was fed into a monochromator (Spex 270M with 100–200 μm slits) and detected with an intensified microchannel plate photomultiplier tube (MCP PMT) and processed with a Becker and Hickl SPC-130 single photon counting system. Fluorescence excitation of the Tb(chelate) and Ru(bpy) system utilized the third harmonic (355 nm) of a diode-pumped, Q-switched Nd:YAG laser (Crystalaser, PL2003), operating at a 10 KHz repetition rate with 5 ns, 10 μJ pulses with a beam diameter of ~2 mm. The fluorescence was collected with a 1 mm diameter optical fiber and directed to a monochromator set to 545 nm with 50 μm slits. The light was detected with a red-sensitive PMT (Hamamatsu R928) and recorded with a 300 MHz Tektronix TDS 3034B oscilloscope, averaging 250 traces. The fluorescence signals from the AuNCs were excited by both laser systems and collected at center wavelengths of 800 nm and 850 nm and averaged over 60–120s. For estimating actual lifetime values, all data were normalized to $t = 0$ (PL maximum) and the lifetimes extracted from only the decay components of the curves.

SQUID (Superconducting quantum interference device) measurement: To check our assumption on the possibility of the existence of paramagnetic species on the surface of our AuNCs we performed magnetic measurements using the Quantum Design Magnetic Properties Measurement System. A mass of 6 ± 0.5 mg of AuNCs was loaded to a Capsugel PCcap® dosing capsule and mounted between two fused silica support tubes and placed within a longer fused silica tube, and suspended from the sample transfer rod. The geometry is similar to that described in previous publication[12] and allows for accurate compensation for sample holder contribution to the magnetization by use of high-purity support material. Measurements of magnetic moment vs temperature (T) were performed on the empty capsule followed by the capsule loaded with AuNC material and the difference considered for analysis using a modified

Curie-Weiss equation to derive the magnetic susceptibility, χ_{abs}; $\chi_{abs} = \frac{C}{T-\theta} + \chi_0$. Here, $C = N\,(g\mu_B)^2 J(J+1)/3k_B$, is the Curie constant, θ is the Weiss temperature, and χ_0 is diamagnetic contribution to the organic matrix that does not play a role in the spin dynamics considered in this work.[13] g is the Lande constant (= 2.0023192778), μ_B is Bohr magneton (= 9.27 × 10-24 J/T), J is the number of spin and k_B is Boltzmann constant (1.3806488 × 10^{-23} m^2 kg s^{-2} K^{-1}).

Quantum Yield Measurements: The quantum yield (Φ) of the AuNC at $\lambda_{em.}$ = 820 nm, Φ_{Au}, in water was determined relative to the known standard, indocyanine green (ICG) ($\lambda_{em.}$ = 800 nm; Φ_{ICG} = 0.13, in DMSO) using Eq. 1. The excitation wavelength was 710 nm. The parameters in Eq. 1 include the integrated PL intensities of the AuNC and ICG in arbitrary units (A.U.), PL_{Au} (1.02 × 10^6 A.U.) and PL_{ICG} (2.46 × 10^6 A.U.), their optical density at 710 nm, OD_{Au} (0.0398) and OD_{ICG} (0.0483), and the refractive indices of their media, n_{Water} (1.333) and n_{DMSO} (1.479), respectively. A similar approach was used to determine Φ for the dyes, metal complexes and the QDs using Rhodamine 6G in methanol ($\lambda_{em.}$ = 566 nm; Φ_{R6G} = 0.93) as the standard:

$$\Phi_{AuNC} = \frac{\int PL_{Au}(\lambda)d\lambda}{\int PL_{ICG}(\lambda)d\lambda}\left\{\frac{OD_{ICG}}{OD_{Au}}\right\}\left\{\frac{n_{Water}^2}{n_{DMSO}^2}\right\}\Phi_{ICG} \qquad \text{(Eq. S1)}$$

Determination of Energy Transfer Efficiency.

I. Donor Luminescence Quenching. The donor PL quenching efficiency, E_Q, was calculated from the ratio of the change in donor fluorescence intensity in the presence of the acceptor to that in the absence of an acceptor:

$$E_Q = \frac{F_0 - F}{F_0} = \frac{(F_0 - F)/\Phi_D^0}{F_0/\Phi_D^0} \qquad \text{(Eq. S2)}$$

where F_0 and F designate the steady-state fluorescence intensities of the donor alone and the donor in the presence of acceptor, respectively.[14] F_0/Φ_D^0, the ratio of the fluorescence intensity of the donor (in the absence of acceptor) to its Φ_D^0, proportional to the total number of photons absorbed by the donor. $(F_0-F)/\Phi_D^0$ is proportional to the total quanta lost through radiative and non-radiative mechanisms. The donor quenching efficiency can also be determined from measured luminescence lifetimes of the donor in the presence and absence of acceptors using:

$$E_{ET} = \frac{Nk_Q}{k_{D0} + Nk_Q} = \frac{Nk_Q}{k_{r0} + k_{nr0} + Nk_Q} = 1 - \frac{\tau_D}{\tau_{D0}} \qquad \text{(Eq. S3)}$$

Where k_{D0} is the sum of radiative decay (k_{r0}) and nonradiative decay (k_{nr0}) components in the absence of acceptors, k_Q (~ k_{ET}) is the quenching rate in the presence of acceptors and N accounts for the number of AuNC acceptor per donor. τ_{D0} and τ_D designate the donor lifetime without and with AuNC (ratio N), respectively.

II. Acceptor Luminescence Sensitization. Energy transfer efficiency can also be determined by measurement of the sensitized luminescence of the acceptor (A-A_o), along with the fluorescence quantum yield of the acceptor (Φ_A^0), where A_0 and A designate the steady-state fluorescence intensities of the acceptor without and with the donor, respectively.[15,16]

$$E_{Sen} = \frac{(A - A_0)/\Phi_A^0}{F_0/\Phi_D^0} \qquad \text{(Eq. S4)}$$

Here, F_0/Φ_D^0 is proportional to the total number of photons absorbed by the donor and $(A-A_0)/\Phi_A^0$ is proportional to the total quanta transferred from donor to acceptor via energy transfer while accounting for the acceptor Φ [15].

Energy Transfer Analysis.

I. Förster Resonance Energy Transfer (FRET): Förster points out in classical theory that the electrostatic interaction energy between two electric dipoles is directly related to the magnitude of the two interacting dipoles and inversely related to the cube of the distance, R, between the donor and acceptor.[14,17-19] Thus, the dipole-dipole energy transfer rate, k_{FRET}, is proportional to R^{-6}. The distance, at which the decay rate of the donor deactivation in the absence of an acceptor, k_{D0}, equals the energy transfer rate from donor to acceptor, is R_0, defined as the critical separation or Förster distance where energy transfer efficiency is 50%.[14,17-19]

$$k_{FRET} = k_{D0}(\frac{R_0}{R})^6 \quad \text{(Eq. S5)}$$

R_0 can be calculated from the donor luminescence and acceptor absorption data using the following equation[14]:

$${R_0}^6 = \frac{9 \ln 10 \; \kappa^2 \Phi_D^0}{128 \, \pi^5 n^4 N_{Av}} J \quad \text{(Eq. S6)}$$

where n is the refractive index of the buffer medium, N_{Av} is Avogadro's number (6.022 × 10^{23} mol^{-1}), $\Phi_D{}^0$ is the donor Φ in the absence of acceptor, κ^2 is the dipole orientation factor, and J is the spectral overlap integral function between donor emission and acceptor absorption. We use a κ^2 of 2/3, which is appropriate for the random dipole orientations of donor and acceptor found within these self-assembled configurations.[14,20] J is determined by integrating the acceptor absorbance $\varepsilon_A(\lambda)$ multiplied by the normalized donor luminescence $f_D(\lambda)$ over all wavelengths, λ. $J(\lambda)$ for each potential donor-AuNC pair can be found in Figure S3.

$$J = \int_0^\infty J(\lambda)\,\mathrm{d}\lambda = \int_0^\infty f_D(\lambda)\varepsilon_A(\lambda)\lambda^4\,\mathrm{d}\lambda \quad \text{(Eq. S7)}$$

If $f_D(\lambda)$ is in nm^{-1}, $\varepsilon_A(\lambda)$ in $M^{-1}cm^{-1}$, and λ in nm, then J is in $M^{-1}cm^{-1}nm^4$ and R_0 (in nm) can be practically calculated using;[14]

$$R_0(\mathrm{nm}) = (\frac{9 \ln 10 \; \kappa^2 \Phi_D^0}{128 \, \pi^5 n^4 N_{Av}} \times 10^{17} J)^{1/6} = (8.79 \times 10^{-11} \kappa^2 \mathrm{n}^{-4} \Phi_D^0 J)^{1/6} \quad \text{(Eq. S8)}$$

Following Eq. S3, the theoretical FRET efficiency from donor to (AuNC) acceptor can be defined as:

$$E_{FRET} = \frac{Nk_{FRET}}{k_{D0}+ Nk_{FRET}} = \frac{Nk_{D0}(\frac{R_0}{R})^6}{k_{D0}+Nk_{D0}(\frac{R_0}{R})^6} = \frac{N}{N+(R/R_o)^6} \qquad \text{(Eq. S9)}$$

where N is the number of AuNCs per donor and R is the center-center distance from donor to acceptor. R was estimated using the molecular size of the ligand, donors and the NC radius as estimated from TEM analysis, see SI for details. If donor quenching were entirely due to FRET, then $E_Q = E_{FRET} = E_{Sen}$. If E_Q does not equal E_{FRET}, then other quenching mechanisms associated with the donor-acceptor complex must be considered.

II. Surface/Volume Damping of Excited Molecules Above a Metal Surface: Energy transfer phenomena from luminescent molecules above a metallic surface were studied extensively by Persson and Lang along with Alivisatos.[21-24] Persson's model, based on Fermi's Golden Rule, calculated transition rates between the dipole of an excited state molecule and a metallic surface for the molecule located at some small distance above a metal surface. De-excitation of the molecule proceeded via interaction between the electric field of the oscillating dipole and the metal surface which was modeled as an electron gas. This occurs simultaneously as an electron in the metal is excited to a state above the Fermi level. Persson suggested two different models to describe the damping rate (energy transfer rate), k_{ET}, of a dye molecule above the metal: (1) Surface Damping, following a ~ $1/R^4$ dependence, involves the excitation of electron-hole pairs in the metal surface, and (2) Volume Damping, which follows a~ $1/R^3 l$ dependence (l = electron mean free path), where the energy is dissipated by conversion of electronic currents in the metal into heat through scattering from phonons, impurities, and other electrons.[21,22] The damping rates were numerically calculated as follows:

$$k_{ET} \approx \frac{\mu^2}{4\hbar}\frac{\omega_D}{\omega_F} F \qquad \text{(Eq. S11)}$$

$$where \qquad F\,(surface) = 1.2\,\frac{1}{k_F R^4}, \qquad F\,(volume) = 3.0\,\frac{1}{k_F R^3 l_{Cor}}$$

here μ is the dipole moment of the dye, R is distance between donor and acceptor (for NSET/NVET, $R = R_S$, distance from the donor to the metal surface), $\hbar$ is the reduced Planck constant, ω_D is the angular frequency of the donor emission, ω_F is the Fermi frequency of the

metal (8.4×10^{15} s^{-1}) and k_F is the Fermi wave vector of the metal (1.2×10^8 cm^{-1}).[21,23] The equations show that volume damping will be dominant for large R and surface damping for small R. For noble metals such as silver and gold l in bulk metal is large at room temperature; l ~430 Å (silver) and 200~250 Å (gold). [25,26] Thus surface damping is normally expected to dominate for $R < 200$ Å for bulk metal.[22] Later, Yun and Strouse applied this surface damping theory to explain the quenching phenomena of dye molecules located near 1.4 nm AuNCs, and coined the term, "Nanosurface energy transfer" (NSET).[27,28] Unlike FRET, these processes do not require a resonant interaction between the surface electrons and the donor dipole, but the damping rate is related to the angular frequency of donor dipole, ω_D.[22]

In the case of metal NPs , however, volume damping may not be negligible because the electron and its mean free path will be confined to the physical size of the NP especially when the NP size is smaller than the traditional mean free path of the electron in bulk metal. Since the volume damping rate was derived by using the dielectric function of the bulk metal,[22] here we suggest that it be adjusted by considering a modified dielectric function with a reduced mean free electron path, l_{Cor}, to account for the nanoscale size of the AuNCs (see the next section). This approach has been repeatedly put forth in the literature, see for example refs.[29-32]

Similar to R_0, the separation distances corresponding to 50% energy transfer efficiency can be derived for both surface and volume damping processes. For surface damping, R_0 corresponding to the separation distance at which energy transfer efficiency equals 50%, was calculated by using the Einstein Coefficient $A21$;[27,33]

$$R_0(Surface) = (0.225\frac{C^3 \Phi_D^0}{\omega_D^2\, \omega_F\, k_F})^{1/4} \qquad \text{(Eq. S12)}$$

where c is the speed of light in vacuum and $\Phi_D{}^0$ is the fluorescence quantum yield of the donor in the absence of acceptor. By analogy, we derive R_0 for volume damping:

$$R_0(Volume) = (0.563\frac{C^3 \Phi_D^0}{\omega_D^2\, \omega_F\;\, k_F\, l_{Cor}})^{1/3} \qquad \text{(Eq. S13)}$$

$$\text{where} \quad l_{Cor} = \left(\frac{2l_0\, l_{eff}}{l_0 + l_{eff}}\right)$$

Here, l_0 and l_{eff} are the mean free path of the electrons in bulk metal and the effective mean free path in NPs when the size of NPs is smaller than l_0, respectively. Thus l_{Cor} is the corrected mean

free path of the electrons in metal NPs (details in SI), which we calculated as ~ 19.2 Å. Based on damping theory, the energy transfer efficiency, E_{ET}, and damping rate, k_{ET} (energy transfer rate), can be described by the expressions;

$$E_{ET} = \frac{Nk_{ET}}{k_{D0} + Nk_{ET}} = \frac{Nk_{D0}(\frac{R_0}{R})^X}{k_{D0} + Nk_{D0}(\frac{R_0}{R})^X} = \frac{N}{N + (R/R_o)^X} \quad \text{(Eq. S14)}$$

$$k_{ET} = k_{D0}(\frac{R_0}{R})^X \quad \text{(Eq. S15)}$$

where X = 4 for surface damping, referred to as NSET hereafter, and X = 3 for Volume Damping, referred to as nanovolume energy transfer or NVET hereafter.

Volume Damping Theory Modification based on Reduced Mean Free Path of Electron

Persson derived the volume damping rate from the bulk metal based on the Drude dielectric function $\varepsilon_{bulk}(\omega)$.[22] In the case of metal NPs, the dielectric function of bulk metal, $\varepsilon_{bulk}(\omega)$ needs to be modified if the metal size is smaller than the mean free path of the free electron in the bulk metal.[29-31]

$$\varepsilon_{bulk}(\omega) = 1 - \frac{\omega_P^2}{\omega_D^2 + i\omega_D\Gamma_0} \quad \text{(Eq. S16)}$$

$$\varepsilon_{NP}(\omega) = \varepsilon_{bulk}(\omega) + \frac{\omega_P^2}{\omega_D^2 + i\omega_D\Gamma_0} - \frac{\omega_P^2}{\omega_D^2 + i\omega_D\Gamma_{NP}}$$

$$= 1 - \frac{\omega_P^2}{\omega_D^2 + i\omega_D\Gamma_{NP}} \quad \text{(Eq. S17)}$$

Γ_0 and Γ_{NP} are the collisional frequency of the bulk metal and the metal NP, respectively. [30,31]

$$\Gamma_{NP} = \Gamma_0 + A\frac{v_F}{l_{eff}}$$

$$= \frac{v_F}{l_0} + A\frac{v_F}{l_{eff}}$$

$$= v_F\left(\frac{l_0 + l_{eff}}{l_0\ l_{eff}}\right)\ (when,\ A = 1) \quad \text{(Eq. S18)}$$

v_F is the Fermi velocity. l_0 and l_{eff} are the mean free path of free electron in the case of the bulk metal and the effective mean free path of the electron in a metal NP respectively. The bulk mean free path of electron of gold is 20~ 25 nm.[25] A is a dimensionless parameter which depends upon the geometry. In the context of simple Drude theory and isotropic scattering, A is usually assumed to be unity.[30,31] However, there is literature that reports A= 0.7 for gold.[34]

Thus, we can derive the volume damping rate for metal NPs by using the reduced mean free path of electrons confined to the size of the NPs following;

$$k_{ET}(Volume) \approx \frac{\mu^2}{4\,R^3\hbar} \times 3.0\ \frac{\omega_D}{\omega_F}\frac{1}{k_F\,l}$$

$$= \frac{\mu^2}{4\,R^3\hbar} \times 3.0\ \frac{\omega_D}{\omega_F}\frac{1}{k_F\left(\frac{2l_0\,l_{eff}}{l_0+l_{eff}}\right)}$$

$$= \frac{\mu^2}{4\,R^3\hbar} \times 3.0\ \frac{\omega_D}{\omega_F}\frac{1}{k_F\,l_{Cor}} \qquad \text{(Eq. S19)}$$

where $l_{Cor} = \left(\frac{2l_0\,l_{eff}}{l_0+l_{eff}}\right)$

l_{Cor} is defined as corrected mean free path of the metal NPs and the value of 2 found within parenthesis is the normalization factor to consider the boundary condition; when the nanoparticle size is close to the bulk mean free path of electron, $l_{eff} \sim l_0$, l_{Cor} reaches l_0.

Thus we can derive the critical distance R_0 from the modified volume damping rate by using the Einstein A21 coefficient.

$$A_{21} = \frac{\omega_D^3}{3\varepsilon_0\hbar\pi c^3}\,|\mu|^2$$

$$R_0(Volume) = (0.563\frac{C^3\Phi_D^0}{\omega_D^2\,\omega_F\ k_F\,l_{Cor}})^{1/3} \qquad \text{(Eq. S20)}$$

If we consider the spontaneous emission is in accordance with Coulomb's law, *1/4πε*, *A21* can be *Φ₀ /4πε*.[27] Here, l_0 and l_{eff} are the mean free path of electrons in bulk metal and the effective

mean free path of electrons in NPs when the size of the NPs are smaller than $l_{0,}$ respectively. And l_{Cor} is the corrected mean free path of electrons in metal NPs. The electron mean free path in NPs was reported to be $l_{eff} = 4V/S$ (V = volume, S = Surface area) for three-dimensional nanoparticles.[31] Also for the sphere, classical theory gives $l_{eff} = r$ (r = radius of sphere) for isotropic scattering, or $l_{eff} = 4/3r$ for diffusive scattering, while the quantum particle in a box model yields, $l_{eff} = 2/3L$ (L = length) for the cube, $l_{eff} = 1.4r$ for a cylinder and $l_{eff} = 1.64r$ for the sphere,[31]. In our calculations that use l_0 = 25 nm and $l_{eff} = 1.64r$ ~ 1.23 nm, l_{Cor} is ~ 2.34 nm.

Decay Rate Constant Calculation.

The total decay rate of the donor, k_{D0} is the inverse of the donor lifetime (τ_{D0}). The radiative and non-radiative decay rates of the donor, in the absence of an acceptor, can be calculated by using the measured donor luminescence lifetime and the quantum yield (Φ_D^0):

$$\Phi_D^0 = \frac{k_{r0}}{k_{r0}+k_{nr0}} = k_{r0}\tau_0 \qquad \text{(Eq. S21)}$$

$$k_{r0} = \frac{\Phi_D^0}{\tau_{D0}} \quad , \quad k_{nr0} = \frac{1-\Phi_D^0}{\tau_{D0}} \qquad \text{(Eq. S22)}$$

Energy Transfer Decay Rate During Donor Quenching

$$E_{ET} = \frac{Nk_Q}{k_{D0}+Nk_Q} = \frac{Nk_Q}{k_{r0}+k_{nr0}+Nk_Q} = \frac{N}{N+k_{D0}/k_Q} \qquad \text{(Eq. S23)}$$

The steady-state donor quenching efficiency can be written (E_T, Eq. 3) by using the donor decay rates before and after quenching. k_Q is the quenching rate and N is the number of AuNCs per donor. k follows a $1/R^6$ dependence (for FRET), or $1/R^X$ dependence (X = 4 for NSET and X = 3 for NVET). [27,28,33,35] As expressed in Eq. 3, the quenching of the energy transfer rate competes with the radiative and non-radiative decay rates. When the quantum yield of donor emission through radiative decay decreases because of energy transfer or quenching, the quantum yield of non-radiative decay will also decrease because of competition with energy transfer, without changing in the intrinsic decay rates, k_r or k_{nr}, themselves. This fact was mentioned by Förster in his earlier paper, "*The efficiency of excitation transfer by resonance is not limited by the fluorescence quantum yield of the donor, because the underlying process is in competition with*

fluorescence emission as well as the other non-radiative processes responsible for fluorescence quantum yields which are lower than unity".[36]

Coupling Parameter Calculation.

In this description for manuscript Equation 3, the resonant energy transfer rate can also be obtained by knowing the coupling parameter between donor and acceptor excited states, V_{DA}, which, in turn, requires knowledge of the donor and AuNC acceptor excited state structure. For the AuNCs, V_{DA} can be estimated from the experimental data. Assuming that the broad AuNC PL band (~250 nm) is associated with the dephasing time, we obtain τ_{cA} ~ 10 fs, which is much shorter than the PL lifetime of AuNCs $\tau_A = 1/\ k_A \approx 0.7$ μs. The values for V_{DA} can then be estimated from the measured donor quenching and AuNC sensitization K_{ET} values for each configuration (2.0×10^4 ~ 3.7×10^7, Table 3) yielding a range for V_{DA} of 1~10 μeV. It is unclear if these estimated values are sufficient for traditional dipole-dipole interactions.

Worm-Like-Chain Model (WLC)

We assumed the PEG as a random coil configuration to determine the size of PEG by solving the mean square end-to-end distance of polymer, $<R^2>$, in worm-like chain (WLC) model (Eq. S24).[37]

$$< R^2 > = 2R_{max}l_p - 2{l_p}^2 \left(1 - \exp\left(-\frac{R_{max}}{l_p}\right)\right) \quad \text{(Eq. S24)}$$

Here $R_{max} = Nb$ is the maximum end-to-end distance of the actual polymer or the length of a fully extended PEG 600 (MW 600 ± 30). N is the repetition number of ethylene glycol units and b is the size of ethylene glycol, 0.15 nm and l_p is the persistence length (stiffness) of the PEG,0.43 nm in aqueous solution.[38] The calculated end-to-end distance of PEG 600 was 1.0 ± 0.15 nm. So we used the average number of R_{max} and end-to-end distance of PEG 600 calculated by WLC model, ~ 1.4 ± 0.15 nm.

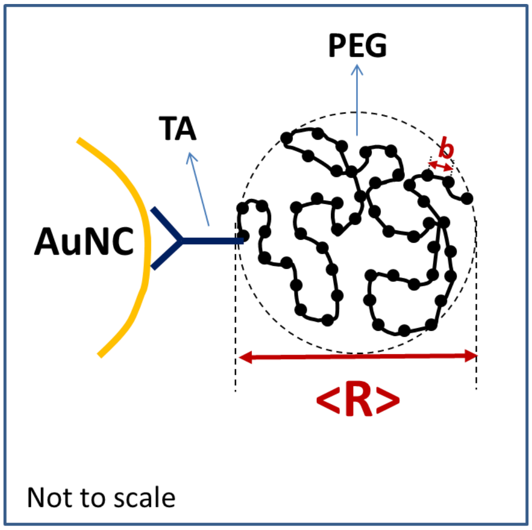

Figure S1. Chemical structures of materials used here. Cy3-NHS, R-Red-NHS, Ru(bpy)-NCS, Tb(chelate)-NCS and Ligands (TA-PEG-NH_2, CL1, MUA)

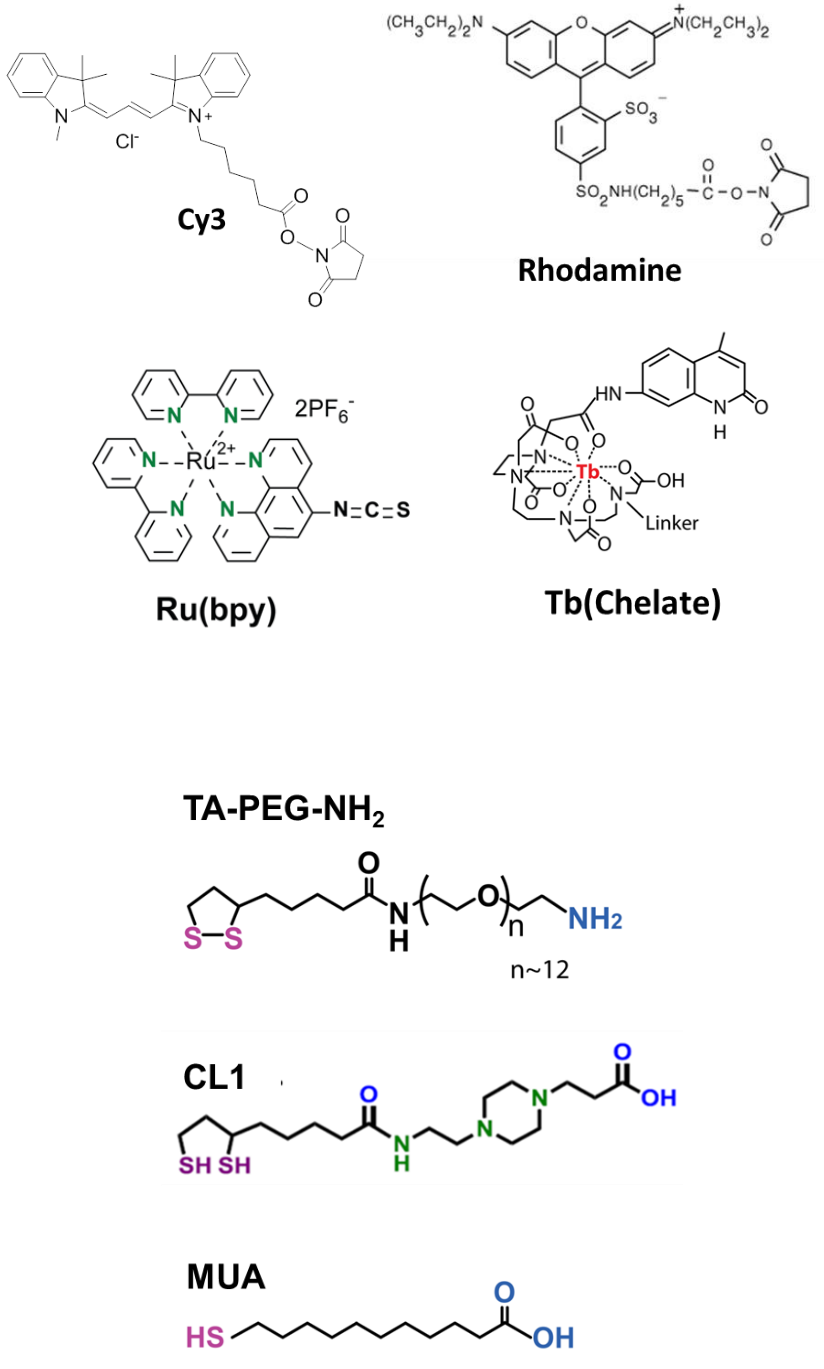

Figure S2. Optical properties of acceptor/donor materials. Absorption spectra (blue line, left axis) and emission spectra (red line, right axis) of acceptor and donors. (a) Luminescent AuNCs. Inset is the excitation spectrum of AuNCs monitored at 820 nm. (b) Cy3-NHS. (c) R-Red-NHS. (d) Ru(bpy)-NCS. (e) Tb(chelate)-NCS. (f) QD545-COOH (absorption: left blue line, 10x scale, emission: left orange line) and QD625-COOH (absorption: right light blue line, 0.2x scale, emission: right red line). Black dotted line in (b) ~ (f) represents the absorption spectra of the AuNCs. We note that both the donor and AuNC acceptor absorption profiles were not significantly altered by incorporation into an AuNC/donor assembly.

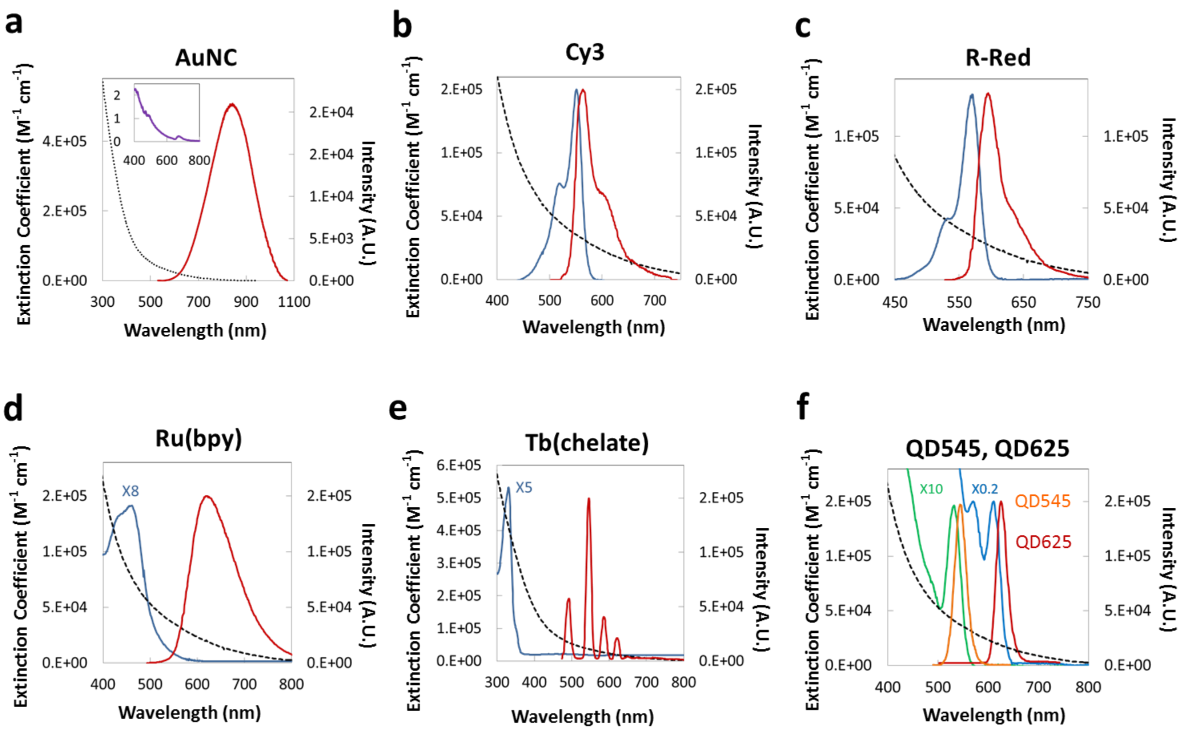

Figure S3. Physical characteristics of AuNC. (A) TEM image. (B) STEM image. The average size is 1.5 ± 0.3 nm. (C) TEM image of QD625. The average size is 9.2 ± 0.81 nm. (D) TEM image of QD545. The average size is 4.0 ± 0.29 nm.

(A)

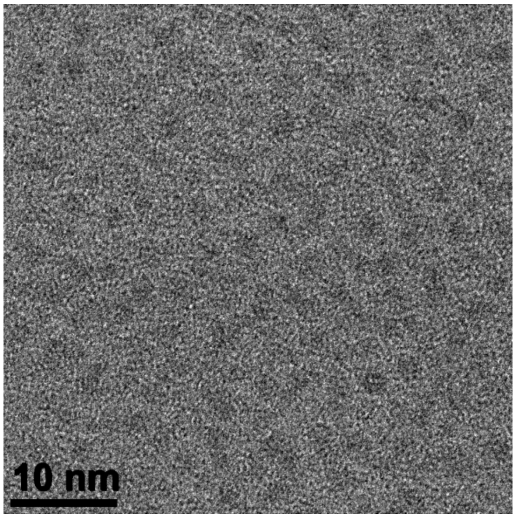

(B)

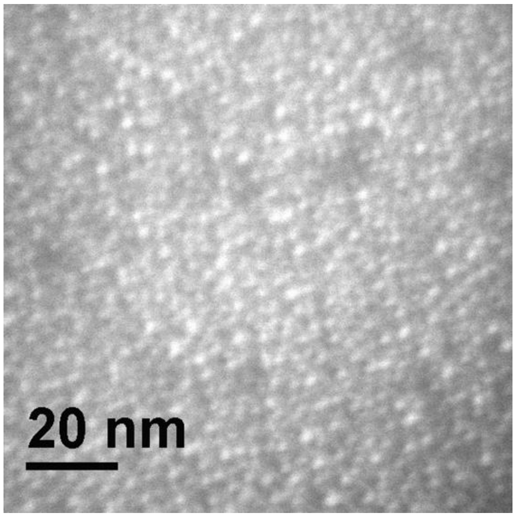

(C)

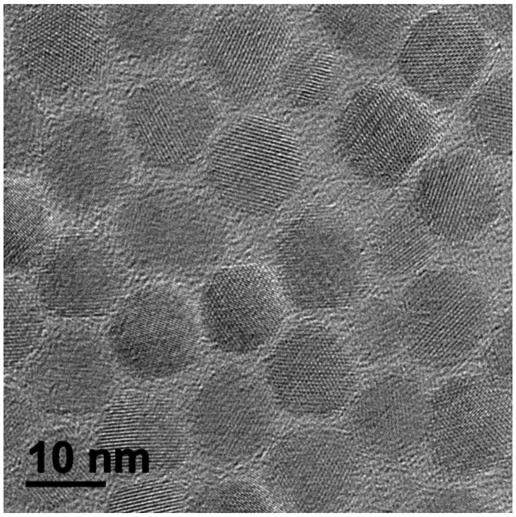

(D)

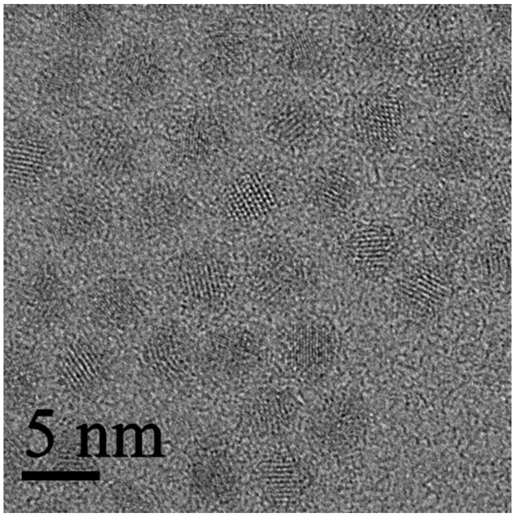

Figure S4. The optical characteristics of AuNCs in various environmental conditions. (A) The absorption spectra of fresh AuNCs along with those that had been stored for several years (> 2 years). The quantum yield of these sample was not dramatically different over time (5 ~ 7% in Water). (B) The absorption spectra of AuNCs in different buffer conditions.

(A)

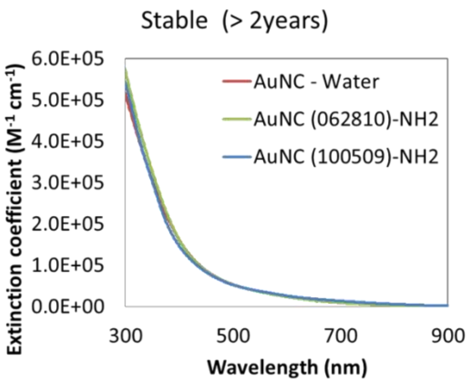

(B)

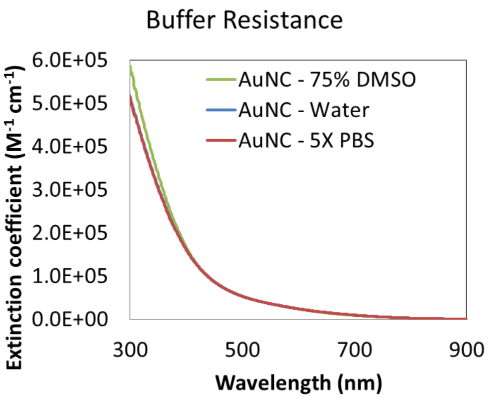

Table S1. TEM, DLS, Zeta-Potential and Mobility Measured for the AuNC and QD625 Samples.

	Z-**Potential** (mV)	**Mobility (μm·cm/Vs)** [a]	**Core Size (nm)**[b]	**Hydrodynamic Size (nm)**[c]	**Theoretical Value (nm)**[d]
**AuNC-NH$_2$**	12.9 ± 1.0	1.01 ± 0.08	1.5 ± 0.3	(8.9 ± 2.2) [e]	6.9 ± 0.37
**QD625-COOH**	-42.1 ± 3.5	-3.3 ± 0.27	9.2 ± 0.8	14.5 ± 3.6	13.2 ± 0.80
**(AuNC)$_{40}$-QD625**	8.7 ± 0.7	0.68 ± 0.06	-	32.4 ± 9.3	27.0 ± 1.10
R[f]	-	-	7.7 ± 0.5	11.7 ± 3.7	10.1 ± 0.44

[a] cm/Vs : Inverse of the applied electric field per cm.

[b] Inorganic core size of each NP and the dried distance between QD and AuNCs measured by TEM.

[c]Hydrodynamic Size: The averaged hydrodynamic size of each sample over number (population) profile and volume (mass) profile of DLS.

[d]Theoretical value: The estimated NP size including ligands based on TEM for inorganic core size and Worm-like chain model for PEG and energy-minimization for stretched size for ligands; CL1 (on the QD625) and TA (on the AuNC).

[e] The calculated hydrodynamic size of AuNC-NH$_2$ based on the hydrodynamic size of QD625 and (AuNC)$_{40}$/QD625 complex system. Using hydrodynamic size values; [(AuNC)$_{40}$-QD625] – [QD625-COOH] = 2 X [AuNC-NH$_2$].

[f] Estimated center-to-center distance between QD625 and AuNC based on TEM (distance after drying ~ 7.7 nm) and hydrodynamic size. Using hydrodynamic size values; [(AuNC)$_{40}$-QD625] – [QD625-COOH] = 2 X [AuNC-NH$_2$]. Then, solve for center-to-center distance, R[f]= [QD625-COOH]/2 + [AuNC-NH$_2$]/2.

Figure S5. The spectal overlap, *J*, for each donor (A) Cy3 (B) R-Red (C) Ru(bpy) (D) Tb(chelate) (E) QD545 (F) QD625

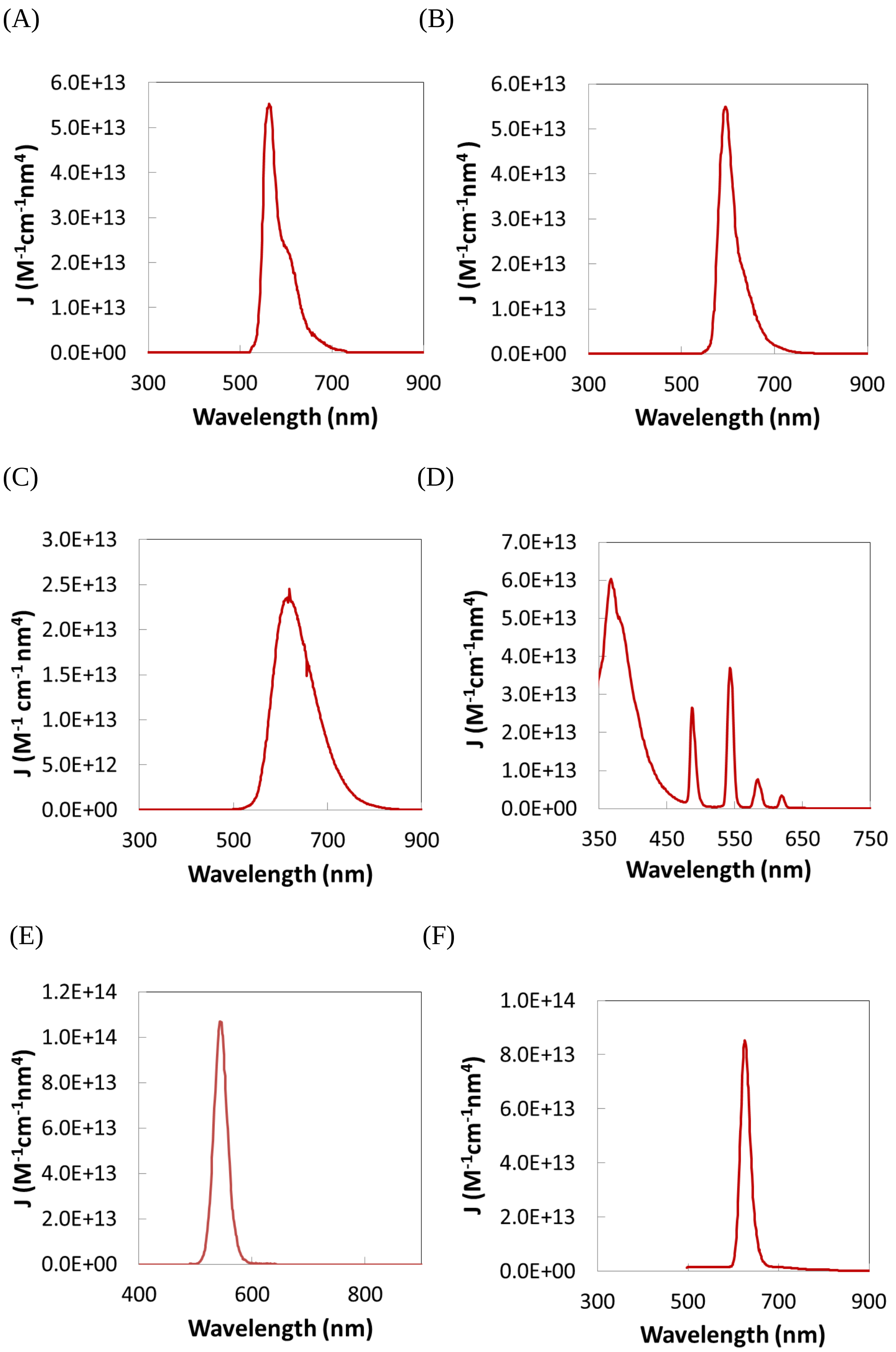

Figure S6. (A) Emission profile of the Tb(chelate) excited at 330 nm. (B) Steady-state quenching efficiencies of the $(AuNC)_N$-Tb(chelate) complexes for each of the prominent emission peaks: 490 nm (E490), 540 nm (E540), 580 nm (E580), 620 nm (E620) and total emission profile (E(Q)). N = 0.08, 0.15, 0.3 and 0.6.

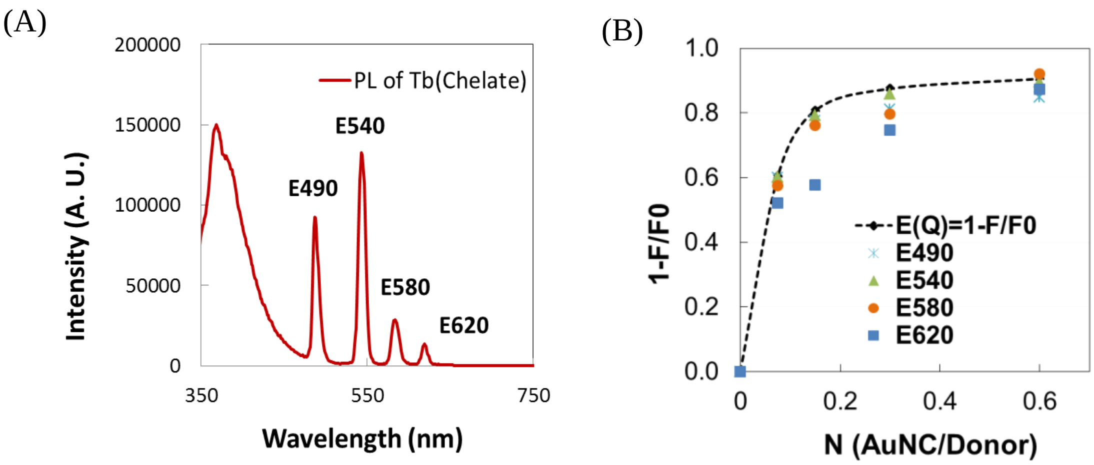

Figure S7. Fluorescence image of $(AuNC)_N$-QD545 complexes with N varying from 0 to 15 under UV lamp illumination (375 nm).

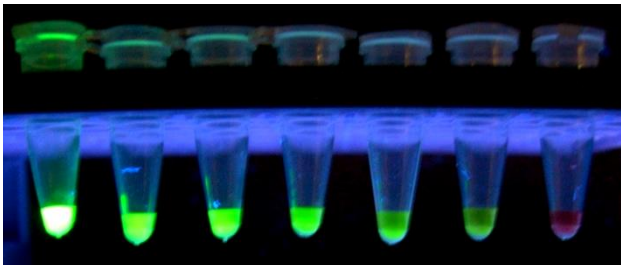

QD545 +Au2 +Au4 +Au6 +Au8 +Au15 Au Only

Figure S8. The spectrum of the 532 nm excitation pulse used for lifetime measurements.

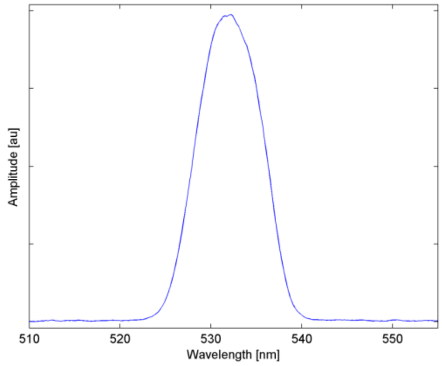

Table S2. Summary of Donor and Acceptor Lifetimes. The numbers in parenthesis next to the lifetimes are the amplitude weight of each lifetime.

Donor	*N*	*Donor Lifetimes* τ_D (Sec)			*Acceptor Lifetimes* τ_A (μSec)		
		τ_{D-1}	τ_{D-2}	τ_{D-Avg}	τ_{A-1}	τ_{A-2}	τ_{A-Avg}
Cy3 –NHS	-	5.7E-10	-	**5.7E-10**	0.98 (68%)	0.19 (32%)	**0.73**
	0.08	7.4E-10	-	**7.4E-10**	0.95 (63%)	0.16 (37%)	**0.66**
532ex,	0.15	6.9E-10	-	**6.9E-10**	0.84 (73%)	0.13 (27%)	**0.65**
580/850em	0.3	7.1E-10	-	**7.1E-10**	0.88 (64%)	0.13 (36%)	**0.61**
	0.6	7.0E-10	-	**7.0E-10**	0.90 (67%)	0.14 (33%)	**0.65**
R-Red-NHS	-	2.8E-9	-	**2.8E-9**	0.98 (68%)	0.19 (32%)	**0.73**
	0.08	2.6E-9	-	**2.6E-9**	0.90 (47%)	0.20 (53%)	**0.53**
532ex,	0.15	2.5E-9	-	**2.5E-9**	1.00 (43%)	0.20 (57%)	**0.54**
580/850em	0.3	2.5E-9	-	**2.5E-9**	0.99 (48%)	0.25 (52%)	**0.60**
	0.6	2.4E-9	-	**2.4E-9**	1.00 (54%)	0.21 (46%)	**0.64**
Ru(bpy)-NCS	-	8.0E-7 (9%)	3.5 E-7 (91%)	**3.9 E-7**	1.40 (45%)	0.20 (54%)	**0.75**
	0.08	6.7E-7 (25%)	3.2 E-7 (75%)	**4.1 E-7**	1.11 (38%)	0.29 (62%)	**0.61**
355ex,	0.15	5.8E-7 (37%)	2.4 E-7 (63%)	**3.7 E-7**	1.03 (56%)	0.12 (44%)	**0.63**
600/800em	0.3	4.3E-7 (47%)	0.6 E-7 (53%)	**2.4 E-7**	1.51 (38%)	0.28 (62%)	**0.75**
	0.6	4.7E-7 (42%)	0.7 E-7 (58%)	**2.4 E-7**	1.69 (37%)	0.30 (63%)	**0.81**
Tb-NCS	-	14.0E-4 (53%)	3.6E-4 (47%)	**9.1 E-4**	1.48 (38%)	0.31 (62%)	**0.75**
	0.08	8.8E-4 (46%)	3.4E-4 (64%)	**5.9 E-4**	1.26 (12%)	0.21 (88%)	**0.34**
	0.15	7.4E-4 (29%)	2.4E-4 (71%)	**3.9 E-4**	1.35 (22%)	0.26 (78%)	**0.50**
355ex,	0.3	10.8E-4 (32%)	1.0E-4 (68%)	**4.1 E-4**	1.42 (34%)	0.32 (66%)	**0.69**
545/800em	0.6	7.7E-4 (29%)	0.2E-4 (71%)	**2.4 E-4**	1.61 (56%)	0.37 (44%)	**1.07**
QD545	-	13.6E-9 (45%)	2.1 E-9 (55%)	**7.3 E-9**	1.39 (50%)	0.27 (50%)	**0.83**
-COOH	2	13.2E-9 (40%)	2.0 E-9 (60%)	**6.5 E-9**	0.85 (65%)	0.17 (35%)	**0.62**
	4	10.8E-9 (29%)	2.4 E-9 (71%)	**4.8 E-9**	0.97 (59%)	0.20 (41%)	**0.65**
532ex,	8	8.6E-9 (27%)	1.9 E-9 (73%)	**3.7 E-9**	1.04 (76%)	0.24 (24%)	**0.85**
550/800em	15	8.1E-9 (32%)	1.8 E-9 (68%)	**3.9 E-9**	1.64 (45%)	0.53 (55%)	**1.02**
QD625-	-	10.6E-8 (50%)	1.7E-8 (50%)	**6.1E-8**	1.24 (52%)	0.19 (48%)	**0.73**
COOH	5	7.0E-8 (35%)	1.5E-8 (65%)	**3.4E-8**	1.08 (50%)	0.24 (50%)	**0.66**
	10	7.2E-8 (43%)	1.5E-8 (57%)	**3.9E-8**	1.27 (48%)	0.25 (52%)	**0.74**
532ex,	20	7.1E-8 (39%)	1.5E-8 (61%)	**3.7E-8**	1.15 (62%)	0.31 (38%)	**0.83**
580/850em	40	6.6E-8 (50%)	1.4E-8 (50%)	**4.0E-8**	1.48 (59%)	0.34 (41%)	**1.01**

Figure S9. Changes in the biexponential lifetime components of the donors of Donor-AuNC conjugations.

A. QD 545

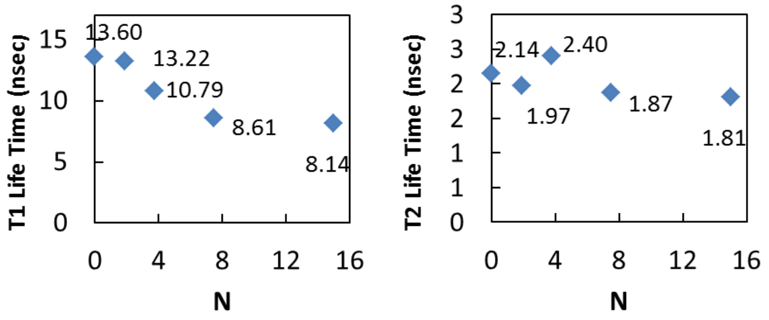

B. QD 625

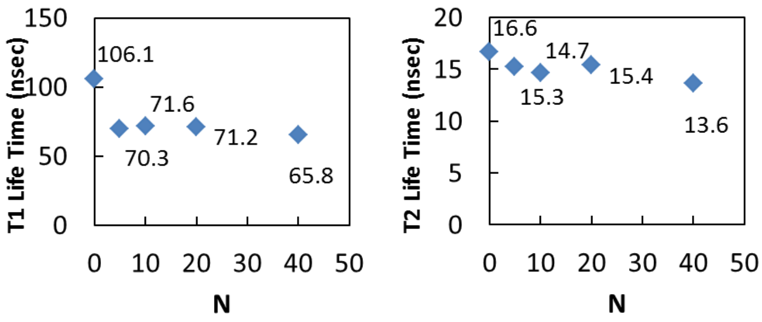

C. Ru(bpy)

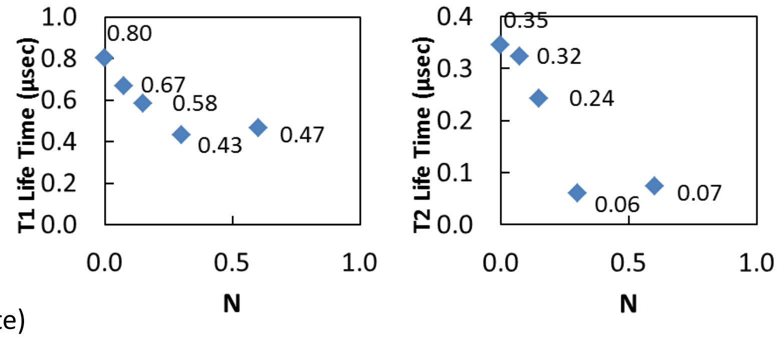

D. Tb(chelate)

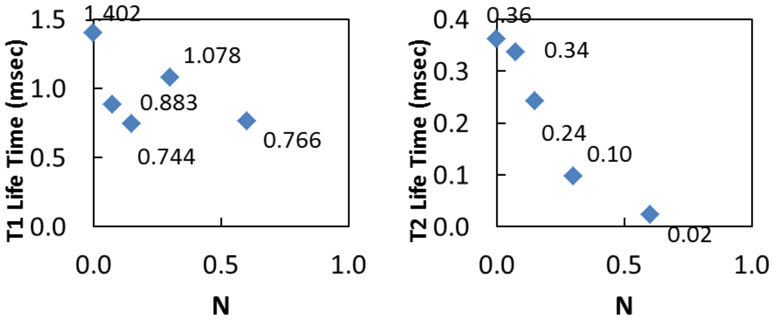

Table S3. AuNC Acceptor and Donor Optical Properties

	ε (M^{-1} cm^{-1})@λ *(nm)*	Angular frequency* ω_D (S^{-1})	Absorption Ratio (compared to AuNC)	AuNC to Donor J ($M^{-1}cm^{-1}nm^4$)
**AuNC**	3.4E+05 @350<br>7.1E+04 @470<br>4.5E+04 @520	-	-<br>-<br>-	
**Cy3**	8.4E+03 @470<br>7.6E+04 @520	3.3×10^{15}	1.5 (*N* = 0.08) ~ 0.12 (*N* = 0.6)<br>21 (*N* = 0.08) ~ 2.8 (*N* = 0.6)	2.5×10^{13}
**R-Red**	1.6E+03 @470<br>3.2E+04 @520	3.3×10^{15}	0.3 (*N* = 0.08) ~ 0.04 (*N* = 0.6)<br>8.9 (*N* = 0.08) ~ 1.2 (*N* = 0.6)	4.1×10^{13}
**Ru(bpy)**	1.6E+04 @470<br>2.8E+03 @520	3.1×10^{15}	2.9 (*N* = 0.08) ~ 0.38 (*N* = 0.6)<br>0.8 (*N* = 0.08) ~ 0.10 (*N* = 0.6)	1.3×10^{13}
**Tb (chelate)**	6.6E+03 @350<br>~0 @520	3.5×10^{15}	0.2 (*N* = 0.08) ~ 0.03 (*N* = 0.6)<br>~0	0
**QD545**	7.1E+04 @470<br>1.2E+05 @520	3.5×10^{15}	0.8 (*N* = 2) ~ 0.10 (*N* = 15)<br>1.3 (*N* = 2) ~ 0.18 (*N* = 15)	1.5×10^{12}
**QD625**	5.9E+06 @470<br>9.1E+05 @520	3.0×10^{15}	16.7 (*N* = 5) ~ 2.1(*N* = 40)<br>4.0 (*N* = 5) ~ 0.5 (*N* = 40)	8.9×10^{14}

*Angular frequency of the emission wavelength.

Table S4. Summary of Experimental and Modeled Energy Transfer Efficiencies

Donor	N (A/D)	Donor Quenching		Acceptor Sensitization	FRET	NSET	NVET
		$1\text{-}F_D/F_0$	$1\text{-}\tau_D/\tau_{D0}$	$\Phi_D\ (A\text{-}A_0)/(\Phi_A\ F_0)$	$N/(N+(R/R_0)^6)$	$N/(N+(R/R_0)^4)^b$	$N/(N+(R/R_0)^3)^c$
**Cy3**	0	0.00	0.00	0.00	0.00 ± 0.00	0.00 ± 0.00	0.00 ± 0.00
	0.08	0.16	-0.29	0.04	0.05 ± 0.02	0.17 ± 0.04	0.44 ± 0.05
	0.15	0.26	-0.20	-0.03	0.09 ± 0.04	0.28 ± 0.05	0.61 ± 0.04
	0.3	0.34	-0.25	-0.16	0.17 ± 0.05	0.44 ± 0.06	0.76 ± 0.03
	0.6	0.41	-0.23	-0.22	0.28 ± 0.08	0.61 ± 0.06	0.88 ± 0.02
**R-Red**	0	0.00	0.00	0.00 ± 0.00	0.00 ± 0.00	0.00 ± 0.00	0.00 ± 0.00
	0.08	0.48 ± 0.01	0.05	-0.15± 0.22	0.05 ± 0.02	0.22 ± 0.03	0.55 ± 0.04
	0.15	0.63 ± 0.05	0.08	-0.54 ± 0.28	0.10 ± 0.03	0.36 ± 0.04	0.71 ± 0.04
	0.3	0.71 ± 0.05	0.11	-0.88 ± 0.39	0.18 ± 0.05	0.52 ± 0.04	0.83 ± 0.03
	0.6	0.74 ± 0.03	0.14	-1.55 ± 0.18	0.30 ± 0.08	0.69 ± 0.04	0.91 ± 0.02
**Ru(bpy)**	0	0.00 ± 0.00	0.00	0.00 ± 0.00	0.00 ± 0.00	0.00 ± 0.00	0.00 ± 0.00
	0.08	0.38 ± 0.17	-0.06	0.37 ± 0.19	0.04 ± 0.02	0.19 ± 0.04	0.47 ± 0.04
	0.15	0.50 ± 0.14	0.05	0.47 ± 0.19	0.08 ± 0.03	0.32 ± 0.05	0.64 ± 0.04
	0.3	0.62 ± 0.10	0.39	0.52 ± 0.18	0.16 ± 0.05	0.49 ± 0.05	0.78 ± 0.03
	0.6	0.74 ± 0.03	0.38	0.60 ± 0.18	0.27 ± 0.07	0.66 ± 0.05	0.88 ± 0.02
**Tb(chelate)**[a]	0	0.00	0.00	0.00	0.00 ± 0.00	0.00 ± 0.00	0.00 ± 0.00
	0.08	0.56 ± 0.04	0.36	0.56 ± 0.02	0.25 ± 0.07	0.46 ± 0.05	0.75 ± 0.03
	0.15	0.76 ± 0.03	0.57	0.76 ± 0.07	0.40 ± 0.09	0.63 ± 0.05	0.86 ± 0.02
	0.3	0.87 ± 0.09	0.55	0.87 ± 0.07	0.58 ± 0.08	0.77 ± 0.04	0.92 ± 0.01
	0.6	0.94 ± 0.03	0.74	0.94 ± 0.02	0.73 ± 0.07	0.87 ± 0.02	0.96 ± 0.01
**QD545**	0	0.00	0.00	0.00	0.00 ± 0.00	0.00 ± 0.00	0.00 ± 0.00
	2	0.29 ± 0.18	0.11	0.23 ± 0.14	0.14 ± 0.03	0.51 ± 0.03	0.87 ± 0.01
	4	0.49 ± 0.17	0.33	0.34 ± 0.10	0.25 ± 0.04	0.67 ± 0.03	0.93 ± 0.01
	8	0.67 ± 0.07	0.49	0.56 ± 0.05	0.39 ± 0.06	0.80 ± 0.02	0.96 ± 0.00
	15	0.83 ± 0.04	0.47	0.93 ± 0.14	0.57 ± 0.06	0.89 ± 0.01	0.98 ± 0.00
**QD625**	0	0.00	0.00	0.00	0.00 ± 0.00	0.00 ± 0.00	0.00 ± 0.00
	5	0.64 ± 0.10	0.44	0.28	0.11 ± 0.03	0.69 ± 0.04	0.96 ± 0.01
	10	0.65 ± 0.12	0.35	0.40	0.20 ± 0.05	0.82 ± 0.03	0.98 ± 0.00
	20	0.63 ± 0.15	0.39	0.63	0.33 ± 0.06	0.90 ± 0.02	0.99 ± 0.00
	40	0.64 ± 0.15	0.34	0.72	0.50 ± 0.07	0.95 ± 0.01	0.99 ± 0.00

[a]Corrected for the cs124 chelate contribution. [b, c] The R and R_0 is the separation and the critical separation between donor and acceptor calculated depending on model.

Increased Energy Transfer Probability From Tb(chelate) to a Single AuNC with Increased Donor /Acceptor Conjugation Ratios.

If the lifetime of the donor is long enough to efficiently transfer energy to the acceptor, the energy transfer probability per single acceptor may increase with increasing donor conjugation number. In multiple donor-acceptor conjugation system, the high chance of energy transfer from the donor to single acceptor will not affect the total energy transfer efficiency of the system (*E*, energy transfer rate per donor), which means that the total energy transfer rate will still decrease with the increased donor conjugation ratio, *M* (=inverse of acceptor conjugation number per donor = *1/N*). However, it changes the probability of that a single acceptor accepts energy from the donor via single energy transfer, as multiple donors are assembled. Thus, single AuNC will become more sensitized and brighter via energy transfer when the number of donors are increased during assembly (as *N* decreases), which can be interpreted as an increase in the net sensitization signal per acceptor, $(A\text{-}A_0)/A_0$ (Eq. S10). The following paragraph is a derivation of the relevant equation.

First, the emission coming from AuNC is the summation of the direct emission from AuNC and the emission via energy transfer from donors in the complex system, with *M (= 1/N)* donors.

$$A = Direct\ Emission\ from\ AuNC + Energy\ Transfer\ From\ Donor$$

Emission is proportional to the total number of absorbed photons by each AuNC (= $C\varepsilon_{Au}$) and the donors (= $C\varepsilon_D\times M$) (assuming the light path length is 1cm). ε_{Au} and ε_D are the extinction coefficient of AuNC and donor, respectively. Since the AuNC-donor complex system has a high absorption (=$C\varepsilon_{Au}$+ $C\varepsilon_D\times M$), the emission from AuNC-donor complex will have contributions from both direct AuNC excitation and energy transfer from the excited donor. Each component, either the AuNC or donor in the complex system, will absorb a fraction of the total number of absorbed photons. Thus the emission from the AuNC in the AuNC-Donor system can be written as follows;

$$A \propto \left\{\frac{C\varepsilon_{Au}}{C\varepsilon_{Au}+MC\varepsilon_D}\times \Phi_{Au}+\frac{MC\varepsilon_D}{C\varepsilon_{Au}+MC\varepsilon_D}\times \Phi_{Au}E\right\}\times (C\varepsilon_{Au}+MC\varepsilon_D)$$

$$= C\varepsilon_{Au}\Phi_{Au}+MC\varepsilon_D\Phi_{Au}E$$

$$= A_0 + \Delta A \qquad \text{(Eq. S25)}$$

Here, E is the total energy transfer efficiency from donor to acceptor in each complex system. C is the concentration of AuNP and thus MC is the concentration of donor. Thus the net sensitization signal will follow,[20]

$$(A - A_0)/A_0 = \frac{MC\varepsilon_D \Phi_{Au} E}{C\varepsilon_{Au}\Phi_{Au}} = M\varepsilon_D E/\varepsilon_{Au} \qquad \text{(Eq. S26)}$$

This equation implies that as the number of donors that surround the acceptor increases (larger M), the emission due to energy transfer from the donor to single acceptor increases and the net sensitization signal per AuNC also increases. Figure S26 presents the net sensitization signal enhancement per AuNC as a function of donor conjugation number, M for our experiments. The most efficient case was the Tb(chelate)-AuNC system and it reached ~ 60% of net sensitization signal enhancement per AuNC (in the case of ~13 donor per acceptor) which reflects Tb(chelate) showed the higher energy transfer probability compared to the other donors.

Figure S10. The net sensitization signal enhancement rate, *(A-A₀)/A₀*, for each donor-AuNC system.

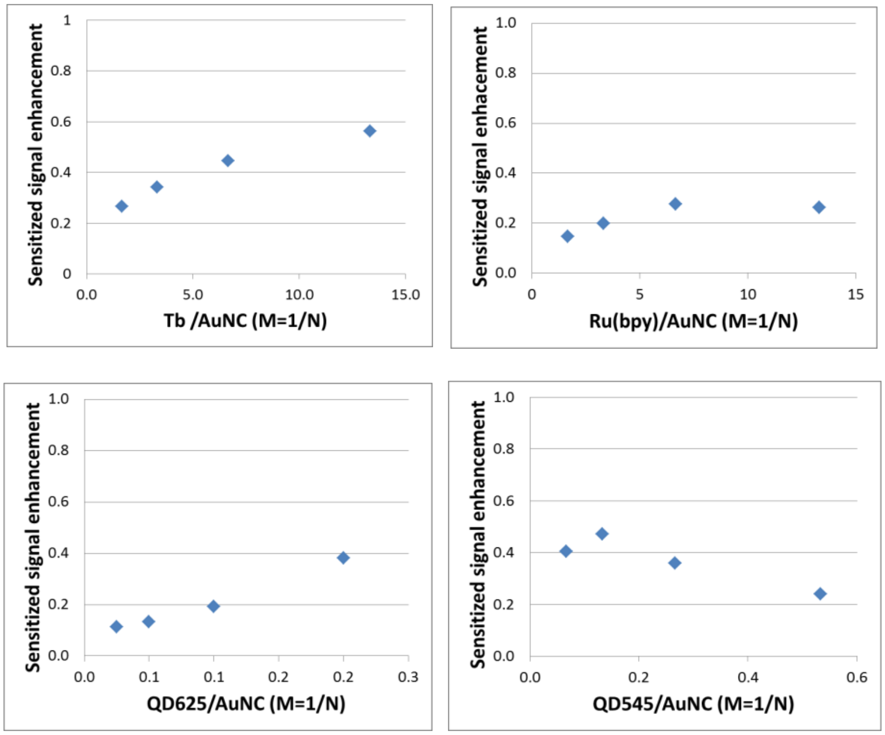

Calculation of Spin Number per AuNC

We consider two different approaches to calculate the molecular weight (MW) of AuNC with 1.5 nm diameter. As common factors, we used the unit volume of Au atom (0.017 nm^3), MW of Au (196.97), MW of ligand (791.048), sample for SQUID measurement (6 mg), and the total spin number measured by SQUID (2×10^{17}).

Figure S11. SQUID measurement from AuNC confirming the existence of paramagnetism due to AuNC unpaired spin.

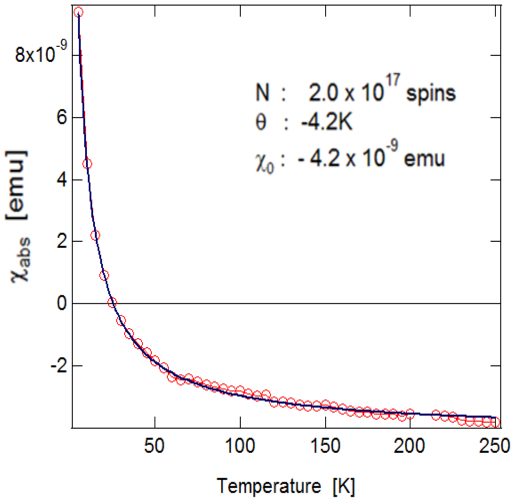

The total number of gold atoms per nanoparticle, NAu and the number of surface gold per nanoparticle, NS-Au based on complete volume filling, can also be calculated using;

$$N_{Au} = \frac{4\pi R^3}{3V_{Au}}$$

(Eq. S27)

Approach 1: Assuming 1.5 nm AuNC as perfect sphere and the thickness of Au shell is 0.238 nm.

$$N_{S\_Au} = \frac{4\pi R^3}{3V_{Au}} - \frac{4\pi\,(R-0.238)^3}{3V_{Au}}$$

(Eq. S28)

where R is the radius of AuNC (0.75 nm) , $V_{Au} \cong 1.7\text{x}10^{-2}$ nm^3 (using an atomic radius of 0.16 nm for Au) and [AuNP] designates the nanoparticle concentration

The calculated total number Au per AuNC was 104, Au number in core was 33, Au number in shell was 71, the number of ligands was ~ 35, and MW of AuNC-NH_2 was 48485, which resulted in the calculated spin number per AuNC ~2.7.

(Approach 2) Assuming 1.5 nm AuNC as perfect sphere and Au 55 as the inner core.

The calculated total number Au per AuNC was 104, Au number in core was 55, Au number in shell was 49, the number of ligands was ~ 24, and MW of AuNC-NH_2 was 39805, which resulted in the calculated spin number per AuNC ~2.2.

Based on crystallographic studies, Jadzinsky reported that similar 1.5 nm mercaptobenzoic acidfunctionalized AuNC's have ≈102 Au atoms in their structure with ≈44 predicted to be on their surface and available for ligand binding.[39] In our bidentate ligands will be 22, MW of AuNC-NH_2 will be 37494, which resulted in the calculated spin number per AuNC ~ 2.1.

Supplemetnary References:

1 Susumu, K. *et al.* Multifunctional Compact Zwitterionic Ligands for Preparing Robust Biocompatible Semiconductor Quantum Dots and Gold Nanoparticles. *Journal of the American Chemical Society* **133**, 9480-9496, doi:Doi 10.1021/Ja201919s (2011).

2 Susumu, K. *et al.* Enhancing the stability and biological functionalities of quantum dots via compact multifunctional ligands. *Journal of the American Chemical Society* **129**, 13987-13996, doi:Doi 10.1021/Ja0749744 (2007).

3 Oh, E., Susumu, K., Goswami, R. & Mattoussi, H. One-Phase Synthesis of Water-Soluble Gold Nanoparticles with Control over Size and Surface Functionalities. *Langmuir* **26**, 7604-7613, doi:Doi 10.1021/La904438s (2010).

4 Oh, E., Susumu, K., Jain, V., Kim, M. & Huston, A. One-pot aqueous phase growth of biocompatible 15-130 nm gold nanoparticles stabilized with bidentate PEG. *Journal of Colloid and Interface Science* **376**, 107-111 (2012).

5 Oh, E. *et al.* PEGylated Luminescent Gold Nanoclusters: Synthesis, Characterization, Bioconjugation, and Application to One- and Two-Photon Cellular Imaging. *Particle & Particle Systems Characterization* **30**, 453-466 (2013).

6 Roberts, M. J., Bentley, M. D. & Harris, J. M. Chemistry for peptide and protein PEGylation. *Advanced Drug Delivery Reviews* **64**, 116-127, doi:10.1016/j.addr.2012.09.025 (2012).

7 Zhang, S. M., Baker, J. & Pulay, P. A Reliable and Efficient First Principles-Based Method for Predicting pK(a) Values. 1. Methodology. *Journal of Physical Chemistry A* **114**, 425-431, doi:10.1021/jp9067069 (2010).

8 Kolny, J., Kornowski, A. & Weller, H. Self-Organization of Cadmium Sulfide and Gold Nanoparticles by Electrostatic Interaction. *Nano Lett.* **2**, 361-364 (2002).

9 Min, Y. J., Akbulut, M., Kristiansen, K., Golan, Y. & Israelachvili, J. The role of interparticle and external forces in nanoparticle assembly. *Nature Materials* **7**, 527-538, doi:10.1038/nmat2206 (2008).

10 Jennings, T. L. *et al.* Reactive Semiconductor Nanocrystals for Chemoselective Biolabeling and Multiplexed Analysis. *ACS Nano* **5**, 5579-5593, doi:10.1021/nn201050g (2011).

11 Stewart, M. H. *et al.* Competition Between FRET and Electron Transfer in Stoichiometrically-Assembled Quantum Dot-Fullerene Conjugates. *ACS Nano* **7**, 9489–9505 (2013).

12 Lewis, L. H. & Bussmann, K. M. A sample holder design and calibration technique for the quantum design magnetic properties measurement system superconducting quantum

interference device magnetometer. *Review of Scientific Instruments* **67**, 3537-3542, doi:Doi 10.1063/1.1147172 (1996).
13 Neil W. Ashocroft, N. D. M. *Solid state Physics*. 1st edn, (Harcourt college publisher, 1976).
14 Lakowicz, J. R. *Principles of Fluorescence Spectroscopy*. (Springer, 2006).
15 Clegg, R. M. Fluorescence resonance energy transfer. *Anaytical Biotechnology* **6**, 103-110 (1995).
16 Hannestad, J. K., Sandin, P. & Albinsson, B. Self-Assembled DNA Photonic Wire for Long-Range Energy Transfer. *Journal of the American Chemical Society* **130**, 15889-15895, doi:Doi 10.1021/Ja803407t (2008).
17 Forster, T. Zwischenmolekulare Energiewanderung Und Fluoreszenz *Annalen Der Physik* **2**, 55-75 (1948).
18 Turro, N. J., Ramamurthy, V. & Scaiano, J. C. *Principles of Molecular Photochemistry : An Introduction*. (University Science Books, 2009).
19 Medintz, I. L. & Hildebrandt, N. *FRET – Förster Resonance Energy Transfer From Theory to Applications.* (Wiley-VCH, Weinheim, Germany 2013).
20 Clapp, A. R. *et al.* Fluorescence resonance energy transfer between quantum dot donors and dye-labeled protein acceptors. *Journal of the American Chemical Society* **126**, 301-310, doi:Doi 10.1021/Ja037088b (2004).
21 Persson, B. N. J. Theory of Damping of Excited Molecules Located above a Metal-Surface. *Journal of Physics C-Solid State Physics* **11**, 4251-4269 (1978).
22 Persson, B. N. J. & Lang, N. D. Electron-Hole-Pair Quenching of Excited-States near a Metal. *Physical Review B* **26**, 5409-5415, doi:DOI 10.1103/PhysRevB.26.5409 (1982).
23 Alivisatos, A. P., Waldeck, D. H. & Harris, C. B. Nonclassical Behavior of Energy-Transfer from Molecules to Metal-Surfaces - Biacetyl(Normal-3-Pi-Star)/Ag(111). *Journal of Chemical Physics* **82**, 541-547 (1985).
24 Avouris, P. & Persson, B. N. J. Excited-States at Metal-Surfaces and Their Nonradiative Relaxation. *Journal of Physical Chemistry* **88**, 837-848 (1984).
25 Kanter, H. Slow-Electron Mean Free Paths in Aluminum, Silver, and Gold. *Phys. Rev. B.* **1**, 522-537 (1970).
26 Schaaff, T. G. *et al.* Isolation of smaller nanocrystal au molecules: Robust quantum effects in optical spectra. *Journal of Physical Chemistry B* **101**, 7885-7891 (1997).
27 Jennings, T. L., Singh, M. P. & Strouse, G. F. Fluorescent lifetime quenching near d=1.5 nm gold nanoparticles: Probing NSET validity. *Journal of the American Chemical Society* **128**, 5462-5467, doi:Doi 10.1021/Ja0583665 (2006).
28 Yun, C. S. *et al.* Nanometal surface energy transfer in optical rulers, breaking the FRET barrier. *Journal of the American Chemical Society* **127**, 3115-3119 (2005).
29 Alvarez, M. M. *et al.* Optical absorption spectra of nanocrystal gold molecules. *Journal of Physical Chemistry B* **101**, 3706-3712, doi:Doi 10.1021/Jp962922n (1997).
30 Averitt, R. D., Sarkar, D. & Halas, N. J. Plasmon resonance shifts of Au-coated Au2S nanoshells: Insight into multicomponent nanoparticle growth. *Phys Rev Lett* **78**, 4217-4220, doi:DOI 10.1103/PhysRevLett.78.4217 (1997).
31 Coronado, E. A. & Schatz, G. C. Surface plasmon broadening for arbitrary shape nanoparticles: A geometrical probability approach. *Journal of Chemical Physics* **119**, 3926-3934 (2003).
32 Link, S., Burda, C., Wang, Z. L. & El-Sayed, M. A. Electron dynamics in gold and gold-silver alloy nanoparticles: The influence of a nonequilibrium electron distribution and the size dependence of the electron-phonon relaxation. *Journal of Chemical Physics* **111**, 1255-1264, doi:Doi 10.1063/1.479310 (1999).
33 Muhammed, M. A. H., Shaw, A. K., Pal, S. K. & Pradeep, T. Quantum clusters of gold exhibiting FRET. *Journal of Physical Chemistry C* **112**, 14324-14330 (2008).

34 Khlebtsov, N. G. Optics and biophotonics of nanoparticles with a plasmon resonance. *Quantum Electronics* **36**, 504-529 (2008).
35 Pons, T. *et al.* On the quenching of semiconductor quantum dot photoluminescence by proximal gold nanoparticles. *Nano letters* **7**, 3157-3164, doi:Doi 10.1021/Nl071729+ (2007).
36 Förster, T. Transfer Mechanisms of Electronic Excitation Energy. *Radiation Research Supplement* **2** (1960).
37 Nedelcu, S. & Slater, G. W. Branched polymeric labels used as drag-tags in free-solution electrophoresis of ssDNA. *Electrophoresis* **26**, 4003-4015, doi:10.1002/elps.200500471 (2005).
38 Kienberger, F. *et al.* Static and dynamical properties of single poly (ethylene glycol) molecules investigated by force spectroscopy. *Single Molecules* **1**, 123-128 (2000).
39 Jadzinsky, P. D., Calero, G., Ackerson, C. J., Bushnell, D. A. & Kornberg, R. D. Structure of a thiol monolayer-protected gold nanoparticle at 1.1 angstrom resolution. *Science* **318**, 430-433, doi:10.1126/science.1148624 (2007).
